# Supplementary material for: A multilingual telephone service for crisis communication with migrant groups: Swedish experiences of responding to the COVID-19 pandemic
Source: BMC Public Health. 2026 Feb 4;26:723. doi: 10.1186/s12889-026-26413-5 (PMC12931007; doi:10.1186/s12889-026-26413-5)
Supplement: Supplementary file 1 — Supplementary Material 1. [file 12889_2026_26413_MOESM1_ESM.docx]

**APPENDIX 1**

Interview guide: Topics

- What type of questions did the telephone service receive?
- How did questions to the telephone service change over time?
- How did HCs manage to answer the questions they received? Were there any questions that they could not answer? How did they deal with these questions/situations?
- What did the HCs think about of the importance of being able speaking their mother tongue with callers?
- What other factors were key to building trust with callers? What other people and organizations have been important for the callers?
- How had callers received information about COVID-19 and vaccination? What type of information seemed to have reached the callers? What type of information seemed to not have reached them?
- What type of information could not be provided on the telephone service that the HCs noticed a need for? What information gaps did they notice in the target group?
- Other experiences on information and COVID-19 and vaccination from the telephone service? What were the HCs’ experiences of organizing the telephone service? Has it been possible to reach the target group with information about the telephone service?
- How did the HCs receive support in order to be able to answer questions on the telephone service?
- What were the HCs’ experiences of being in contact with civil society in order to reach out with information about COVID-19 and vaccination?
- What lessons learned do they want to highlight? Other experiences?
