# Supplementary material for: A multilingual telephone service for crisis communication with migrant groups: Swedish experiences of responding to the COVID-19 pandemic
Source: BMC Public Health. 2026 Feb 4;26:723. doi: 10.1186/s12889-026-26413-5 (PMC12931007; doi:10.1186/s12889-026-26413-5)
Supplement: Supplementary file 2 — Supplementary Material 2. [file 12889_2026_26413_MOESM2_ESM.docx]

**Title: A multilingual telephone service for crisis communication with migrant groups: Swedish experiences of responding to the COVID-19 pandemic**

**Authors:** Sofie Bäärnhielm, Baidar Al-Ammari, Önver Cetrez, Soorej Jose Puthoopparambil, Mattias Strand

**Keywords:**

COVID-19, communication, language, culture, health crisis, pandemic plan

**Corresponding author:** Sofie Bäärnhielm

Email: [sofie.baarnhielm@ki.se](mailto:sofie.baarnhielm@ki.se)

Address:

Transcultural Center, Region Stockholm

Solnavägen 1e floor 7, 113 65 Stockholm, Sweden

**ABSTRACT**

**Background:** Migrants living in socioeconomically disadvantaged neighborhoods in Sweden were overrepresented among the infected and deceased in coronavirus disease 2019 (COVID-19) and vaccination coverage was substantially lower, despite being free of charge. The overarching aim of this study was to learn from the experiences of operating a multilingual telephone service for public health crisis communication targeting migrant communities in Sweden during COVID-19. An additional aim was to achieve an in-depth understanding of opportunities and challenges in communicating up-to-date information about COVID-19 and vaccination to lay persons.

**Methods:** A qualitative design based on in-depth interviews with 12 health communicators staffing the telephone service was used. Additional quantitative descriptive data on the use of the telephone service are provided for context.

**Results:** The quantitative data revealed that relatively few callers requested basic information about the virus or asked about topics such as where to turn in case of illness. The most common topic was testing for current infection. The thematic analysis identified seven major themes: The need for a multilingual telephone service; the interaction between language, culture, and profession for building trust; successful knowledge transfer; the importance of cooperation with other actors and organizations; managing existential concerns, emotions, and mental distress; replying to false information and myths; and lessons for a telephone service in the event of a new health crisis.

**Conclusions:** For dissemination of information about COVID-19 and vaccination to migrants during the pandemic, experiences from the multilingual telephone service point to the value of communication that includes the possibility of dialogue with health professionals in a culturally safe mode using one's native language. For future health crises, our findings emphasize the importance of having a communication strategy targeting vulnerable groups in place as a part of a comprehensive pandemic plan when the need emerges.

**Trial registration:** The study protocol has been preregistered on the Open Science Framework (osf.io/rt47j)

**BACKGROUND**

“No-one is safe until we are all safe”— the words of World Health Organization (WHO) Director-General and the mantra of the WHO during the coronavirus disease 2019 (COVID-19) pandemic—point to the global importance of ensuring that all groups in a society are being protected from the virus [1]. A crucial component in reaching this goal is accurate and effective public health crisis communication about the virus and the recommended precautions. For multicultural societies, this includes the challenge of reaching and establishing a dialogue with diverse sociocultural, linguistic, and religious groups. The COVID-19 pandemic has revealed problems at individual, group, and structural levels and highlighted the importance of inclusive health communication strategies that reflect the needs and contexts of various population subgroups in a society.

In Sweden, migrant groups living in socioeconomically disadvantaged neighborhoods were overrepresented among the infected and deceased [2–5]. For the entire duration of the pandemic in Sweden, a substantially higher risk of COVID-19 intensive care unit admission was seen among migrants from Africa, Middle-East, Asia, South America, and other European countries compared to Swedish-born individuals [6]. Similar patterns were seen for mortality in COVID-19 among groups with different geographical origin. Moreover, despite the fact that COVID-19 vaccination was free of charge and implemented with the ambition of reaching the whole Swedish population above 15 years of age, vaccination coverage in Sweden was substantially lower in the same socioeconomically disadvantaged multicultural areas that were most severally hit in the first pandemic wave [7].

These findings in the Swedish context mirror those observed on a global scale. Racial and ethnic disparities in COVID-19-related morbidity and mortality, not least affecting refugees and asylum seekers, were reported from multiple countries early on in the pandemic [8–10]. In retrospect, it is evident that the COVID-19 pandemic disproportionately affected migrant communities worldwide, largely due to structural inequality, residential segregation, and barriers to healthcare [4,11,12]. Individuals with a migration background tend to more often be employed in the service sector, where working from home is seldom an option, or in healthcare and care for the elderly, where the risk of acquiring and/or transmitting infections may be higher [13]. Cramped housing accommodation and a tendency for more socializing across generations might also have contributed to higher burden of disease [11,13]. Moreover, migrants who are new to a country may find it difficult to navigate the healthcare system, which becomes an obstacle in comprehending medical information [14,15]. Importantly, migrant populations typically had relatively low vaccine uptake once COVID-vaccines became available, the reasons for which included limited access, stigma, and vaccine hesitancy [12,16–19].

Information from public authorities that is poorly adapted to the particular needs of migrant groups has been suggested as another important contributing cause behind the observed patterns [20,21]. Official recommendations need to be adapted to real-life conditions in order to be seen as understandable, relevant, and trustable. For example, working from home or avoiding public transportation is unfeasible for certain groups in society, such as many refugees and migrants, for whom it may be more useful with hands-on guidelines on how to best handle cramped living conditions [22,23]. Population groups that perceive official recommendations as unrealistic or irrelevant to their particular situation risk feeling less valued as citizens, becoming alienated, and putting less trust in government agencies [24].

Furthermore, knowledge of the local language may be poor, especially among elderly migrants and those who have newly arrived in a country [15]. Some may also have to rely on verbal rather than written information due to illiteracy. As reported on a global level as well as in Sweden, a significant proportion of refugees and migrants relied on news from their country of birth as the main source of information about COVID-19 [23,25]. This may have had a negative impact on the knowledge of local recommendations and restrictions.

Importantly, several of these mechanisms may interact in giving rise to feelings of exclusion, alienation, and lack of trust. In the public health work of government agencies, ‘cultural competence’ as well as ‘structural competence’ are therefore necessary components of any successful intervention. The concept of cultural competence can be defined as healthcare professionals’ gradually developed capacity to provide safe and high-quality healthcare to patients of different cultural backgrounds, with defining attributes such as cultural awareness, cultural sensitivity, cultural knowledge, cultural skills, and an emphasis on *becoming* rather than *being* culturally competent [26]. Structural competence in healthcare, in turn, refers to the ability to recognize how health is affected by broad social, political, and economic structures, including healthcare systems, food availability, local infrastructure, zoning laws, etc. [27]. Importantly, cultural and structural competence are vital components in identifying barriers to patient *agency*—which can be defined as a socio-culturally mediated capacity to act [28]—and self-determination in health behaviors and healthcare, especially so for marginalized groups [29].

Two factors contributed in making intercultural health crisis communication a particularly pressing issue in Sweden during the early stages of the COVID-19 pandemic. First, Sweden has a relatively large migrant population compared to many other European countries. Foreign-born persons currently make up 20.4% of the Swedish population; when including persons born in Sweden to two foreign-born parents, the number is 26.8% [30]. Second, contrary to most other countries, Sweden did not enforce a strict lockdown policy during the pandemic. Instead, Swedish authorities opted to rely primarily on voluntary public health measures for mitigating the spread of COVID-19, on the grounds that this strategy would be more sustainable [31]. The Swedish pandemic approach proved to be a controversial choice that required well-measured communication efforts from governmental agencies, not least towards groups in society that tend to rely less on ‘mainstream media’ and more on foreign news outlets and social media [25]. Notably, at the outset of the COVID-19 pandemic, the newly updated epidemic preparedness plan of Region Stockholm [32] did not include any measures to reach various local population groups.

**A multilingual telephone service**

To reach non-Swedish-speaking migrant communities in the greater area of Stockholm, the Transcultural Center—the public resource center for migrant health in Region Stockholm—took the initiative to launch a regional multilingual telephone service for questions concerning COVID-19 and vaccination in April 2020 [33]. In June 2020, this telephone service was nationalized in order to serve the whole country, in cooperation with the Public Health Agency of Sweden (PHAS). It operated until April 2022. The telephone service did not offer individual-level medical advice; instead, its primary purpose was to answer questions and queries of a more general character about COVID-19 and vaccination in minority languages. The service was open between 9 AM and 3 PM on weekdays. Callers could choose between a number of minority languages: Arabic, Amharic, Dari/Farsi, English, Russian, Serbo-Croatian, Somali, Spanish (available intermittently), and Tigrinya.

The national telephone service was staffed by multilingual health professionals, most of whom were so-called health communicators, who were employed by Region Stockholm or Region Östergötland (a region in southeast Sweden), although they answered calls from the whole country. The HCs are individuals with a migration background of their own [32]. They all have an education in medicine or public health; a typical background of a HC may be someone who worked as a nurse or a medical doctor in their country of origin, but who has not yet been able to validate their diploma after arriving in Sweden. Before the pandemic, the HCs worked in local contexts within a public health intervention aimed to increase personal health literacy and facilitate help seeking among migrants. This work involved meeting newly arrived refugees and migrants living under socially precarious and stressful conditions. In their work with the multilingual telephone service, the HCs worked from home with weekly digital team meetings and advisory meetings with the PHAS and Smittskydd Stockholm (the regional infection control authority), addressing up-to-date recommendations and offering support on how to answer new or difficult questions.

The telephone service was funded by Region Stockholm, Region Östergötland, and the PHAS. It was promoted through established governmental and regional information outlets (such as official websites, advertisements in newspapers and public transportation, etc.). There were also additional promotional efforts in close collaboration with various partner networks, including civic society organizations who regularly meet migrant groups.

**Theoretical framework and aims**

This study builds on the theoretical framework for health crisis communication outlined by the United States Centers for Disease Control and Prevention (CDC) and others, emphasizing the phased, situation-specific, and culturally sensitive nature of effective outreach and community engagement with vulnerable populations [34,35]. This framework is centered around the necessity of building *trust* (i.e., individual- and community-level confidence in the competence, fairness, transparency, and accountability of risk management leadership) in times of crisis. This capacity is, in turn, influenced by crisis psychology—acknowledging that people tend to process and act on information differently under stress than they would in non-crisis contexts [34]—and can be strengthened by iterative communication strategies that actively engage vulnerable populations and acknowledge their cultural orientations, priorities, and lived realities [35]. Notably, ‘hotline’ telephone services, such as the one described here, are listed by the CDC as a typical examples of a high-level community engagement activity.

The overarching aim of this study was to learn from the experiences of establishing and operating a multilingual telephone service for public health crisis communication targeting migrant communities during the COVID-19 pandemic, in order to strengthen and improve the capacity for intercultural health communication during future health crises. An additional aim was to achieve an in-depth understanding of opportunities and challenges in communicating up-to-date information about COVID-19 and vaccination to lay persons during the pandemic. We also hoped to contribute some critical reflections on the topics of agency and communication in a context of COVID-19 in Sweden.

**METHODS**

**Study design**

For this study, a qualitative research design based on several individual in-depth interviews and one focus group interview with health communicators (HCs) staffing the telephone service was employed. Moreover, in order to provide some additional context, quantitative descriptive data from the telephone service are presented.

**Data collection and analysis**

Quantitative data

Descriptive data on the multilingual COVID-19 telephone service were collected and documented in survey-format by the PHAS from June 1, 2020 until April 30, 2022. These survey data were entered in real-time by the health professionals staffing the telephone service and included the regional location of the callers, the preferred language used, the specific topics raised during the calls, and whether the callers were referred to the government-run national healthcare telephone service “1177”, operated by nurses and other healthcare professionals. An optional free-text entry for further information was also available. No external validation of the staff’s data entry was performed. The data were accessed with permission from the PHAS; however, any information that could potentially compromise confidentiality was deleted by the PHAS. A descriptive statistical analysis was performed utilizing IBM® SPSS® Statistics version 28. Missing data were excluded from the analysis.

Qualitative data

An interview guide was created, covering the following areas: Experiences of organizing the telephone service; the type of questions received; how questions changed over time; experiences of answering questions; perception of the importance of language; and the perception of key factors to establishing trust and report with callers (see Appendix 1). These areas were chosen based on the identification on three domains of language barriers, cultural issues, and structural issues outlined above as well as on the findings in the quantitative component. To ensure maximum relevance, the interview guide was designed with active input from one of the HCs who had staffed the telephone line and who participated as a researcher in the project. The included items were phrased so as to assure informants that their individual performances were not being evaluated and that the aim of the interviews were to identify overarching themes that might guide similar crisis response interventions in the future.

The sole criterion for inclusion in the qualitative interviews was that informants should have staffed the multilingual telephone service during the pandemic. No explicit exclusion criteria were employed. Individual in-depth face-to-face interviews were conducted with six of the seven HCs in Region Stockholm that staffed the telephone service; the seventh HC participated as a researcher in the project and was not interviewed. These interviews lasted between 30 and 90 minutes. An in-person focus group interview lasting 90 minutes was conducted with the six HCs from Region Östergötland. All interviews were performed in Swedish. Since the qualitative interviews included all of the HCs staffing the telephone service in Region Stockholm and Region Östergötland (except for the HC participating as researcher), an almost full coverage was achieved. In order to mitigate any informant bias (such as a potential tendency to exaggerate the benefits and success of the telephone service), the interviews explicitly focused on areas of potential improvement for future health crises. An overall impression is that the informants did not express any concern in voicing potentially ‘uncomfortable’ opinions and that the interview data contained a vast array of potential areas of improvement.

All interviews were digitally recorded and transcribed verbatim. The transcripts were anonymized by omitting or changing details that could risk identifying persons who had contacted the telephone service.

A thematic analysis framework was used in the analysis of the interview data [36]. From an epistemological perspective, this framework reflects a view of knowledge and meaning as created through social interactions and experiences that are necessarily interpreted through the lens of cultural, contextual, and individual-level factors. Our analytical approach was explorative and inductive and thus not based on pre-established theoretical conceptions [37]. First, all authors familiarized themselves with the interview data. Two authors (BAA and SB) then coded the data together, identifying meaning units and developing preliminary codes, categories, and themes. These were discussed and revised in an iterative process involving both coders, until a preliminary interpretation of the content and categorization of the meaning units were agreed on. This interpretation was then discussed with all authors, who participated in a final revision of the themes and subthemes.

**Ethics and preregistration**

This study was conducted in accordance with the ethical standards of the Helsinki Declaration of 1975, as revised in 2008. The study was approved by the Swedish Ethical Review Authority (No. 2022-01637-01). Written consent was obtained from all participants. The study protocol has been preregistered on the Open Science Framework (osf.io/rt47j).

**RESULTS**

**Quantitative data**

There were, in total, 9 414 calls to the telephone service. Of these calls, 4 150 were registered and documented in survey-format by the PHAS. The most commonly requested languages were Arabic (33.8%), English (29.8%), Persian/Dari (14.9%), Tigrinya (9.6%), and Russian (5.6%). Few callers requested information in Somali (2.5%), and even fewer in Amharic (1.1%), Serbo-Croatian (<1%), and Spanish (<1%).

A large majority of callers brought up only one or two topics (74.0% and 20.1%, respectively). The single most common topic was polymerase chain reaction (PCR) testing for *current* COVID-19 infection, raised in 39.6% of all calls. Fewer calls concerned the topic of antibody testing for *past* COVID-19 infection (3.6% of all calls). Another very common topic was how to acquire a travel certificate after a PCR test (26.0% of all calls). The distribution among languages was fairly equal for these topics.

In 14.7% of all calls, the question about when and how to get vaccinated was raised. Notably, of all calls in Tigrinya, 33.0% (132 calls) concerned this topic. Calls about vaccine safety and side effects were few (2.6% and 2.3%, respectively). Here too, calls in Tigrinya stand out, having raised this topic in 12.0% and 7.0% of all calls, respectively. Only 5.3% of all calls concerned the topic of *why* to get vaccinated, with no large differences between languages. Also noteworthy, calls about travel certificates after vaccination were much less common (2.5% of all calls) than calls about travel certificates after PCR testing.

Of all calls, only 5.0% brought up the topic of where to turn in case of COVID-19 illness. However, this topic was raised in 17.4% of all calls in Somali, in 9.9% of all calls in Russian, and 9.4% of all calls in Arabic. Overall, few callers requested basic information about what the corona virus is (0.9%), how the virus is spread (1.2%), at-risk groups (1.4%), how to avoid becoming infected (1.3%), how to avoid infecting others (2.1%), or how to access more information about the corona virus (3.7%).

In 16.0% of all calls, other topics than those listed above were raised. The free-text entries provided in the PHAS data sheet indicate that some of these calls concerned practical issues, such as how to book a vaccination appointment without electronic identification, how to get tested without having a national social security number, vaccination appointments for asylum seekers, etc. Having had a first vaccine dose in a foreign country was also raised in a number of calls. Other topics concerned quarantine, participation in outdoor sports, and concern about public facilities not following recommended guidelines.

In total, 56.6% of all calls resulted in a referral to the national healthcare telephone service “1177”. Notably, 87.5% of Somali-speaking callers were referred to “1177”.

With the quantitative results indicating the migratory background of the callers and the common questions asked, we now move on to the qualitative results that provide an in-depth understanding on how the HCs experienced the calls, the challenges they faced and how they addressed them.

**Qualitative data**

The thematic analysis identified seven major themes: 1) The need for a multilingual telephone service; 2) the interaction between language, culture, and profession for building trust; 3) successful knowledge transfer; 4) the importance of cooperation with other actors and organizations; 5) replying to false information and myths; 6) managing existential concerns, emotions, and mental distress; and 7) lessons for a telephone service in the event of a new health crisis.

Theme 1: The need of a multilingual telephone service

This theme deals with the HCs’ perception of the need for a multilingual telephone service and their experience in setting it up and adapting it over time. At the beginning of the pandemic in particular, the HCs experienced a great need for information in their native languages. There was a significant spread of infection in migrant-dense areas, while there was a lack of multilingual governmental information. Many callers had some limited knowledge of Swedish, but not on the level required for understanding the somewhat technical and complex information from the authorities. The HCs also highlighted the importance of verbal dialogue made possible by the telephone service, as some migrants are illiterate.

The type of questions the HCs got changed as the pandemic progressed. In the beginning, they mostly received questions about virus transmission, protection against infection, symptoms of COVID-19, testing, and how to access medical emergency treatment. Later on, they received more questions about the COVID-19 vaccines. However, callers did not only have questions about COVID-19. Many questions were about personal social and economic consequences of the pandemic. These questions were more challenging for the HCs to answer, since it was not part of their professional task. Many callers posed questions related to their own health issues and currently experienced symptoms. The HCs had neither the mandate nor the expertise to answer these types of medical questions. Instead, they were supposed to refer callers to the official Swedish healthcare telephone service, “1177”. The fact that the HCs could not answer medical questions caused some frustration among callers, prompting comments such as:

*“Why do you run the telephone service if you can't help us”.*

In sum, there was an obvious need for verbal information provided in the native languages of migrants throughout the pandemic. However, the ‘moving target’ character of the pandemic as well as questions related to personal medical and socioeconomic issues proved challenging for the HCs.

Theme 2: The interaction between language, culture, and profession for building trust

The importance of building trust with callers was emphasized by the HCs. Their impression was that the combination of a shared language, knowledge of different cultures and contexts, and the status of the HCs as being a part of the Swedish healthcare system contributed in establishing trust. Building trust was an active process during the conversation with callers:

*“Usually, you started with something—with a question—and then when they felt reassured they would ask, ‘okay, can I ask something else, it may not be relevant but I think you are the right person to ask these questions to’.”*

The HCs emphasized that many of the callers came from countries and communities where people generally trust neither the authorities nor the healthcare system:

*“In many countries you don't trust the healthcare system, you don't trust, well, the people who run the country. And many times there is some relevance to it… So many of these people we meet are people who don't trust the system.”*

The special significance of being able to use one’s native language in the pandemic situation, in order to fully grasp the situation and to avoid misunderstanding official governmental information, was highlighted by the HCs. This included the possibility to ask clarifying questions related to health issues that might be taken for granted by government agencies:

*“And also the difference between: What is a virus, what is a bacterium? What do [these terms] really mean, you know?”*

Furthermore, the cultural background of the HCs and their emic (i.e., in-group) insight into as well as their lived experience (subjectivity) about the callers’ sociocultural context were seen as crucial in being able to fine-tune the response and, accordingly, to building trust:

*“I could offer support, I could facilitate, both by knowledge and by how… And there is a difference if you say ‘do this, drink hot water!’. But if you can become more personal, you know—for example, knowing that Afghans make a lot of green tea, that you relate to something that you know this person can also relate to and know that ‘okay, do this, do that!’."*

Another important factor in building trust through credibility was the professional competence of the HCs and their clear affiliation with the healthcare system. Moreover, the fact that the HCs could offer the callers hands-on support in how to get tested for the virus and book a time for vaccination contributed to building trust:

*“There was no shame in asking [us]. Many people could not make an appointment if they wanted a COVID test. When the testing began, we helped the person to download the [digital ID], ‘this is how to do it’, and they got help step by step.”*

In sum, establishing trust among callers was an active and perhaps somewhat counterintuitive process that involved the HCs’ language skills and sociocultural sensitivity as well as their formal status as affiliated with the Swedish healthcare system.

Theme 3: Successful knowledge transfer

This theme refers to the HCs' perception that they could contribute knowledge that was received and understood by the callers and that seemed to have a real impact on their behaviors. For successful knowledge transfer, the HCs stressed the importance for callers to get a chance to ask questions and discuss, instead of relying solely on one-way information.

Many times, the HCs received feedback from callers showing that they were really able to disseminate knowledge about COVID-19 and vaccines to them and that this had real-life consequences. One example was when callers considered getting vaccinated:

*“This person had read a lot about it on social media [exclaiming]: 'This is how it is, you give the wrong information.' Finally, he accepted. He called me twice. But he accepted [and told me]: ‘Yes, I will get vaccinated’.”*

When the HCs perceived that they succeeded in transferring knowledge to the caller, they got the impression that this information was also being passed on to other individuals in the community. Callers would mention that they intended to tell others, such as family, friends and other acquaintances, about what they had learned. When callers were clearly skeptical about vaccination, it was important that the HCs offered them plenty of time to talk. The HCs described how even those who were initially quite firm in their beliefs could eventually change their minds. Some of those who contacted the telephone service ended up calling several times. The ability to call repeatedly was seen as important for conveying new messages about COVID-19 and vaccines. This possibility meant that the caller had the opportunity to reflect, check multiple sources of information, and ask new questions.

It was important for the HCs to answer credibly even to personal questions. This became a concern especially at the beginning of the vaccination program, when they were asked to explain why they themselves had not been vaccinated:

*“In the beginning it was a bit difficult, when the vaccine arrived and we hadn't been offered the first dose. ‘Oh, you're not a good role model, you have to get vaccinated first’. Questions like that. One time I was asked, ‘Have you been vaccinated?’. They started vaccinating the older ones and I was waiting for my age [group to be offered the vaccine]. ‘Do you think you will get vaccinated?’ ‘You have to get vaccinated before you talk about vaccines!’”*

One challenge that hampered knowledge transfer for the HCs was that the Swedish pandemic strategy differed markedly from those of other countries. Callers were aware of different guidelines being implemented in their countries of origin and the HCs needed to take the time to discuss this. Furthermore, callers often expressed confusion related to the Swedish approach. One HC put it this way:

*“Sweden had slightly different guidelines compared to other countries, and if people watch [television] channels from their home countries and they see these very strict rules, with face masks, lockdown, no one going to school, then there’s a lot of confusion among the callers about who is right and who is wrong.”*

When this topic came up, the HCs did not try to convince the callers about the Swedish pandemic strategy; they simply explained what the guidelines were and the official rationale behind them. They also urged callers to turn to government sources for information.

In their dialogue with migrants, the HCs also highlighted behavioral change as a way of showing consideration for others. One HC referred to discussions with callers who did not want to get vaccinated as they considered themselves to have an excellent immune system:

*“But we told them about the importance of taking responsibility for others too. You get vaccinated for your own sake, but also to protect your loved ones and everyone else in the community.”*

In sum, the opportunity for dialogue, as opposed to one-way, top-down communication, was emphasized as a main strength in enabling successful knowledge transfer.

Theme 4: The importance of cooperation with other actors and organizations

Effective collaboration with other organizations in promoting the telephone service was stressed by the HCs. Extensive efforts were made to reach community-level organizations that could be assumed to be in contact with the target groups of the telephone service. Cooperation with the civic society was considered important not only to make the telephone service known to the public, but also to deliver a common message about COVID-19 and vaccination. Cooperation with religious communities was seen as particularly helpful. Religious leaders and the messages they sent were perceived as having a significant impact on people's attitudes towards COVID-19 and vaccination. The HCs used their out of office hours and their networks to provide information on COVID-19 and vaccination, outside of the telephone service:

*“I have spread the information in churches and left brochures in shops. I usually do it in my spare time, leaving brochures with health information and information about the telephone service. In this way, I have also been in contact with the housing agency to disseminate information.”*

Other important actors that the HCs cooperated with were schools teaching Swedish for migrants, educational associations, and housing companies.

Collaboration with religious communities was also essential for countering false information and myths (discussed in more detail below). However, the HCs also experienced occasional difficulties when religious leaders contributed to spreading false or misleading perceptions. One HC described the importance of religious leaders based on a conversation about vaccines, where the view of the vaccine as a “tool of the devil” came up. For the most part, however, religious communities were supportive in disseminating accurate information.

Theme 5: Replying to false information and myths

Working with the telephone service sometimes involved replying to false information and popular myths about the origin of the virus, virus transmission, and the vaccines that circulated in the society. Although many callers were clearly influenced by misinformation, none were considered as fully rigid in these opinions. The callers were always perceived to have at least some form of curiosity which enabled a meaningful dialogue. Some appeared to call to learn about arguments that could be used in discussions with others and to muster the courage to take the vaccine themselves:

*“… there were those who had some doubts and wanted some kind of reassuring confirmation that it is not dangerous and that it is the best remedy and that the myths that flourish or are being spread were not true.”*

However, there were challenges for the HCs in how to handle false information and myths about COVID-19 and the vaccine. The callers referred to many different sources of COVID-19 information, and sometimes to an explicit lack of information. These sources included friends and family, religious communities and leaders, and social media. Many referred to information from their countries of origin. The HCs described that fear was clearly a motivation for callers engaging with misinformation. Thus, being open to addressing peoples’ fears proved important for questioning myths and misconceptions:

*“Many times it is not easy, because they fear for their lives and are very convinced. But the fact that they call is a positive thing. If you're absolutely determined and don't want to be vaccinated, for example, then you don't call.”*

An approach that was found to be successful in responding to false information and myths was to clearly anchor your arguments in scientific knowledge. For example, a common doubt among callers was related to the perception that the vaccine had been developed too quickly and that there would be potential unknown consequences of getting vaccinated. In these calls, the HCs raised the importance of being critical of the sources of information:

*“In those cases, we stood on firm ground where we could refer to science as long as we had the information and [could point to] reliable sources.”*

In sum, misinformation was abundant during the COVID-19 pandemic, but the HCs were generally able to find ways to counteract myths and misconceptions through active dialogue and by adhering firmly to established evidence.

Theme 6: Managing existential concerns, emotions, and mental distress

This theme reflects how questions about COVID-19 were often posed in a narrative context involving feelings of worry and fear, social hardship, personal difficulties, and loss. The HCs gave many examples of talking with callers who expressed fear about virus transmission and their own health. Other topics of concern included social and economic consequences of the pandemic, and the wellbeing of friends and relatives in their countries of origin. It was challenging for the HCs to deal with the strong emotional reactions they encountered. One HC described how callers would talk about death, one of the core existential concerns, in terms of the loss of family members and friends in their countries of origin:

*“Those who had lost their loved ones to COVID in Iran. Yes, I had a lot of those calls, it was very bad.”*

Fear among callers made it more challenging to convey information. Notably, several calls to the telephone service revealed other existential concerns related to loneliness, anxiety, and isolation, especially at the beginning of the pandemic. The regular health-related support systems were disrupted:

*“[T]hey were feeling very bad and isolated. In our culture, when you are sick, people come and visit you. But in this case, during the pandemic, everyone was afraid to see each other and therefore the loneliness affected them a lot, they felt bad mentally. Many people ask us where they can turn.”*

Sometimes callers did not have an actual question but primarily seemed to need someone to share their worries with. To support the callers, the HCs would then simply listen to them:

“*Mostly, it was that they wanted to talk. Because [here] they actually had the chance to talk. Sometimes you had to say that there were other calls waiting, but they just wanted to talk. It was difficult—the night is long when one has COVID […]. They get stressed and it's hard for us to talk for a long time, but sometimes I used to give them time and talk to them.”*

The HCs had previous work experiences of encountering refugees and migrants in difficult situations involving mental distress. Their approach was to guide people to resources on mental health, while not themselves trying to act as mental health professionals or counselors. There was a fine line between offering guidance and getting overly involved. The HCs received support from the PHAS and Smittskydd Stockholm in the form of regular meetings, educational sessions, and access to rapid assistance regarding new questions. However, this revolved mostly around issues about the COVID-19 virus and the vaccine, and not so much around how to address any existential, emotional, and mental difficulties encountered in callers. To a large extent, the HCs were left on their own in dealing with emotional calls, which was sometimes perceived as difficult.

Theme 7: Lessons learnt for future health crises

This theme focuses on the lessons that the HCs articulated for operating a telephone service in the case of a pandemic in the near future, but also on lessons concerning the communication of health-related information in a more general sense. The need for the telephone service became urgently apparent at the beginning of the pandemic. Therefore, a lesson for the future was to make sure to quickly set up a similar multilingual telephone service in the event of a new pandemic. The HCs stressed the importance of this service also being culturally appropriate.

A limitation that the HCs experienced was that they could not provide medical advice. For a future telephone service during a pandemic or a similar health crisis, they wanted to include staff who are mandated to provide case-appropriate medical advice. Another limitation was that the HCs experienced a need for better support concerning the emotional problems and mental distress they encountered in many of the callers. The HCs also wished they could have provided better psychological support themselves.

In Sweden, healthcare is run by the regional governments. Yet another challenge was the problem of operating a nationwide telephone service in a healthcare system in which different Swedish regions applied different regulations and routines. For example, the specific routes to testing and vaccination would often differ between regions. Callers typically needed hands-on guidance on what to do and where to turn, and the HCs found it difficult to keep track of the various procedures in different parts of the country.

In the event of a future pandemic, the HCs felt that a telephone service was particularly important for people who are new to the country. Newly arrived migrants experience unique difficulties with the Swedish language and with navigating the healthcare system and making sense of the information provided by government agencies.

In sum, multilingual telephone services similar to the one evaluated here can be potentially helpful in future health crises. To ensure optimal effectiveness, future interventions should accommodate for a broader range of needs among callers, including psychological distress and medical referrals.

**DISCUSSION**

In this study, the quantitative data and the qualitative findings complement each other in ways that we will discuss in more detail below. The analysis of the quantitative data describing the calls made to the multilingual COVID-19 telephone service revealed that relatively few callers requested basic information about the virus or asked about topics such as where to turn in case of illness. A hypothetical reason behind this is that the telephone service was less well-known in the early phase of the pandemic, when these issues were potentially most urgent. In contrast, the more commonly asked questions about vaccination became relevant in a later phase, when the telephone service was more well-established.

Notably, Somali speakers made up only 2.5% of callers, but when they did call, they more often requested information on where to turn in case of illness compared to other groups. Moreover, 87.5% of all calls in Somali were referred to the national healthcare telephone service “1177”. This indicates that this group might have needed personal medical advice more often than other groups and that their condition may have been worse once they did call. Individuals born in Somalia (together with those born in Iraq and Syria) were clearly overrepresented in terms of excess mortality during the Spring 2020 compared to Swedish-born individuals [2]. As in previous epidemics, the COVID-19 pandemic quickly gave rise to alarmist media representations of what has been called “the unsanitary other” [38] as driving the spread of infection. In Sweden, the Somali-speaking group in particular were affected by these negative media representations. Interviews with Swedish-Somali youth in a socioeconomically underprivileged neighborhood in Stockholm highlight feelings of enormous injustice associated with “being blamed for your own death” while struggling to uphold physical distancing in a context of overcrowded housing and precarious jobs [39].

The theme “The need of a multilingual telephone service” from the qualitative analysis points to the importance of lay people being able to use their native language when accessing information about a health crisis. Furthermore, the HC experiences underscore the need to allow for dialogue-based communication where questions can be asked and discussed in a relatable context. Importantly, public health messages to minority groups in a pandemic cannot simply be offered in the form of literal translations of the information provided in the dominant language. Instead, health messages targeting these groups must address relevant social determinants of health and reflect the social, cultural, and linguistic lived realities of minority populations [20]. Moreover, making relevant information available in multiple languages is not only about ensuring that the message is understood, but also about signaling the importance of societal inclusion during a health crisis [40].

The theme “Successful knowledge transfer” gives insight into the HC experiences of the importance of dialogue. This is consistent with the emphasis on a dialogue-based communication approach to successfully address vaccine hesitancy and resistance [41]. Effective public health communication involves an emphasis on ‘communicating with’ rather than ‘communicating to’, so that those communities that government agencies wish to reach are also allowed to become engaged as stakeholders in designing and optimizing communication strategies. Importantly, this approach acknowledges the existence of agency among the most vulnerable. In recent years, the importance of identifying ‘blind spots’ in mainstream public health campaigns has increasingly been stressed, in order to better tailor health communication with vulnerable groups that may not share underlying assumptions or find conventional health messages relevant to their contexts [42]. This paradigm shift has occurred against a backdrop of conventional public health communication in which health risk behaviors have typically been framed as individual and moral choices, often resulting in victim blaming and stigmatization [43,44]. Unfortunately, while public health advocates often point to the need to address sociocultural determinants of health, governments still tend to favor policy actions that aim to change individual-level behaviors through social marketing [45]. This is in spite of the known fact that people generally do not engage in health risk behaviors due to a lack of knowledge about risk, but because of life constraints that make them unable or unwilling to act differently [46]. More recently, previously accepted one-way models of health communication have been challenged, in favor of strategies that aim to uncover everyday contextualized experiences of health and illness and engage communities in a cooperative manner [47]. In the context of the multilingual telephone service, allowing callers to voice their own thoughts and opinions with someone with similar lived experiences of migration to Sweden helped establishing a reciprocal mode of communication.

The theme “The interaction between language, culture, and profession for building trust” addresses the HC experiences of the significance of actively building trust with callers. The HCs found that trust was established through a combination of shared language, emic knowledge of culture and context, and their status as being a part of the Swedish healthcare system. In a sense, the HCs’ role in the work with the multilingual COVID-19 telephone service resembles that of ‘cultural brokers’, i.e., go-betweens mediating and translating between culturally distinct spheres [48]—in this case, this involves linguistic spheres as well as the different spheres inhabited by healthcare experts and lay people.

The theme “The importance of cooperation with other actors and organizations” addresses the importance of local collaborative work. Although the telephone service was national, the HCs worked to reach and engage with local civic society organizations. Religious organizations were considered particularly important. Such collaboration should take the form of a two-way exchange, as described above, and may include sharing resources and facilities as well as working together to tailor culturally and structurally relevant information to local groups. Local religious leaders have proven to be valuable partners in disseminating information on COVID-19 restrictions and vaccination during the pandemic in other settings [40,49,50]. Ethnic minority congregations often have a longstanding history of mobilizing local resources and acting like safe havens in times of crisis [51]. Of course, there are also examples of religious leaders contributing in spreading false information and myths during the COVID-19 pandemic. If anything, this underscores the importance of engaging in dialogue with local religious institutions [52].

Another lesson to be learned from the multilingual telephone service is the importance of ensuring access to continuous and up-to-date scientific support by relevant government agencies in a health crisis. It has been noted that handling a ‘mega-crisis’ such as the COVID-19 pandemic inevitably involves navigating unknown territories and adapting to new and quickly changing circumstances [53]. The COVID-19 pandemic saw a rapid development of scientific knowledge and frequent changes in guidelines and recommendations from authorities. This was not least evident during the early phase when the multilingual phone service was launched. Access to the best available scientific support, in the form of regular dialogue meetings with relevant government agencies, was crucial in keeping pace with the “moving target” of the pandemic. Even so, an undue emphasis on the COVID-19 pandemic as uncharted waters may ignore the fact that several tried-and-tested public health interventions simply were not in place at the beginning of the pandemic. This includes, for example, a lack of pre-established collaborations with local civic society representatives to optimize efforts for reaching and disseminating information to migrant groups. The HCs staffing the multilingual COVID-19 telephone service thus had to combine the known-but-not-implemented and the new and unknown in their work.

The theme “Managing existential concerns, emotions, and mental distress” includes the important finding that the callers to the multilingual telephone service did not merely pose factual questions about COVID-19 and vaccination. Many calls were made in an obvious state of fear about the virus and its medical and social consequences, leaving the individual in a state of conflict between ultimate questions of life and death [54]. Sometimes, callers would not even ask a question but simply express a need to vent their worries. The immediate impact of the COVID-19 pandemic on population-level mental health may have been slightly exaggerated [55]; even so, a meta-analysis suggests that there might have been more than a threefold increase in the rates of anxiety in the general population during the pandemic [56]. For survivors of COVID-19, persistent psychological problems including depression and anxiety have frequently been reported [57]. Among the reported risk factors for the development of anxiety were social isolation, unemployment, financial hardship, low education level, and insufficient knowledge of COVID-19. Furthermore, a low level of satisfaction with or trust in the measures taken by the government correlated with high anxiety scores. Telephone crisis support workers may experience vicarious traumatization, stress, and burnout, and they may not respond optimally to callers when experiencing elevated levels of distress [58]. In order to safeguard their well-being, the staff needs basic skills in crisis management as well as the opportunity to access support and guidance of their own. These findings echo those of other studies pointing to the importance of support for mitigating stress reactions among healthcare staff during future pandemics [59].

The theme “Replying to false information and myths” address HC experiencing how the COVID-19 pandemic has brought about a massive parallel ‘infodemic’ of false and unsourced information [60,61] This includes poorly substantiated theories about the origin of COVID-19 and the peddling of fake cures. Several theories on how to prevent the spread of misleading health information and ‘nudge’ people to make healthy behavior changes have been put forward. For example, so-called ‘inoculation models’ highlight the preemptive spread of scientifically sound information and the promotion of critical thinking [62]. One specific approach that the HCs found to be successful for responding to what they identified as misinformation was to firmly anchor the discussion in available scientific knowledge. As far as the HCs could tell from return callers, the possibility to discuss with a knowledgeable healthcare professional in a culturally safe mode using one’s native language proved fruitful for reaching community members with limited trust in government information.

The theme “Lessons learnt for future health crisis” includes not relying solely on digital information in a future health crisis. The strong current tendency in Swedish healthcare to rely on the internet and social media for the dissemination of health-related information risks becoming somewhat of a double-edged sword. In an evaluation of the handling of the COVID-19 pandemic, the Swedish Association of Local Authorities and Regions points to recently arrived immigrants as a group that has been exposed to digital exclusion, due to factors such as a lack of knowledge of the Swedish language and limited digital ability [63]. Similarly, our quantitative and qualitative data both show that a substantial number of calls concerned practical issues related to online healthcare solutions, such as how to book a vaccination appointment without electronic identification. Although a telephone service may seem like an old-fashioned option, many vulnerable groups do not have full access to newer online communication channels. The existence of individuals who rely on verbal information due to illiteracy is also often overlooked.

Combining quantitative and qualitative findings allows for some further conclusions. Based on the quantitative descriptive results, a majority of calls were conducted in Arabic and English with fewer calls in in Persian/Dari, Tigrinya, Russian, Somali, Amharic, Serbo-Croatian, and Spanish. Even so, there were no obvious discrepancies in terms of staff narratives based on the languages spoken; i.e., HCs expressed similar experiences and concerns regardless of the specific language services they provided. This points to shared structural barriers rather than language- and culture-specific issues among callers as the most common reasons for contacting the telephone service.

In sum, the qualitative findings underscore the importance of offering an opportunity for dialogue in building trust in authorities’ public health information. These findings are in line with the theoretical framework for health crisis communication outlined by the CDC and others, emphasizing the phased, situation-specific, and culturally sensitive nature of effective outreach and community engagement with vulnerable populations [34,35]. More specifically, the HC experiences point to a lack of trust early on in the COVID-19 pandemic—in part related to common crisis psychology, but also aggravated by a lack of readily available culturally adapted information—that they felt that they were subsequently able to mitigate in their work with the multilingual telephone service. One interpretation of our findings is that while language and cultural competency were instrumental in building trust in the HCs staffing the telephone service, the actual questions posed by the callers were often related to structural barriers. This is reflected in the results from a study looking at direct and indirect effects of the COVID-19 pandemic on multiple social groups in Sweden, showing that the relative risk of being affected by negative events were strikingly similar across groups during the pandemic as in the four years preceding it [64]. In effect, the socials determinants of health that disproportionally affected vulnerable groups in society before the pandemic were also the most important factors at play during the COVID-19 pandemic. This also means that there is not necessarily a need to reinvent the wheel. Culturally and structurally relevant health communication efforts that actively involve marginalized populations can clearly contribute to reducing the impact of health disparities, during a health crisis as well as in non-crisis times.

**Strengths and limitations**

The HCs participating in this study all had almost two years of experience in staffing the multilingual COVID-19 telephone service, enabling a unique insight into the possibilities and challenges associated with operating a crisis communication intervention for migrant groups. The aim of this study was to gain an unbiased insight into HC experiences of operating the telephone service and there was a great openness among the informants to share their experiences.

The findings presented here must also be viewed in light of a number of limitations. For example, the study is based on the experiences of 12 HCs only. However, these 12 HCs had answered most of the 9 414 calls to the telephone line. It should also be noted that a qualitative study does not require a specific sample size in order to produce meaningful results [65]. In terms of the trustworthiness and rigor of the qualitative analysis, the fact that the HC experiences were based on a large number of telephone calls from all over Sweden covering almost the entire period of the pandemic supports the credibility of the findings. The thorough analysis process involving five researchers who reviewed the meaning units, themes, and subthemes several times until consensus was reached, as well as the use of the NVivo software, were ways to address rigor and reliability [66]. Another weakness is that quantitative data on calls that concerned questions of a personal nature were removed by the PHAS for reasons of confidentiality, limiting our possibilities to draw more detailed conclusions regarding worries related to personal medical issues and referrals to the national healthcare telephone service “1177”. An overall limitation is that the nature of the available data does not allow us to analyze precisely which groups were actually reached by the telephone service and which were not, beyond the statistics on the preferred language of the callers. Yet another limitation is that it was not possible to use a prospective study design, since the multilingual telephone line was set up in an emergency pandemic situation. Finally, although the study was not designed to evaluate the performance of the HCs, there is a possibility that some staff experiences might not have been shared for fear of reflecting poorly on the telephone service.

**Conclusion and implications**

Experiences from a multilingual telephone service in Sweden point to the value of health crisis communication that offers the possibility of dialogue with health professionals in a culturally safe mode using one's native language. The results highlight the importance of linguistic, cultural, and structural competence for communicating and disseminating relevant information and building trust in times of a health crisis. Moreover, for future research, the experiences from this study point to the necessity of making use of the (often retrospective) data that are possible to collect in a naturalistic pandemic emergency setting. Our findings emphasize the importance of having a communication strategy that targets vulnerable groups in place before the need arises, as part of a comprehensive pandemic plan. This is particularly important for linguistically and socioculturally diverse communities in which the uptake of public health information created with the majority population in mind cannot be taken for granted. Moreover, the ability of marginalized groups to navigate the healthcare system can be strengthened by establishing dialogue and cooperation with civic society organizations and other local representatives of migrant communities. Sufficient support structures providing up-to-date scientific information must be available. However, health crisis communication interventions such as a telephone service should also be able to accommodate emotional reactions, existential concerns, and mental distress among callers, and not only straightforward factual questions. The HC experiences have the potential to contribute important knowledge about vital aspects of multilingual crisis communication relevant to settings wider than a pandemic.

**List of abbreviations**

CDC: United States Centers for Disease Control and Prevention

COVID-19: Coronavirus disease 2019

PCR: Polymerase chain reaction

PHAS: Public Health Agency of Sweden

RR: Relative risk

WHO: World Health Organization

**Ethics approval and consent to participate**

The study was approved by the Swedish Ethical Review Authority (No. 2022-01637-01). Written consent was obtained from all participants.

**Consent for publication**

Not applicable.

**Availability of data and materials**

The datasets used during the current study are available from the corresponding author on reasonable request.

**Competing interests**

The authors declare that they have no competing interests.

**Funding**

The current study is funded by the Swedish Research Council (reference number 2021-06276).

**Authors’ contribution**

Sofie Bäärnhielm: Conceptualization; Data curation; Formal analysis; Funding acquisition; Investigation; Methodology; Project administration; Resources; Software; Supervision; Validation; Writing - original draft; Writing - review & editing.

Baidar Al-Ammari: Data curation; Formal analysis; Investigation; Writing - review & editing.

Önver Cetrez: Conceptualization; Investigation; Methodology; Writing – review & editing.

Soorej Jose Puthoopparambil: Conceptualization; Investigation; Methodology; Visualization; Writing - review & editing

Mattias Strand: Conceptualization; Data curation; Formal analysis; Investigation; Methodology; Writing - original draft; Writing - review & editing

**Acknowledgments**

We want to thank the participating health communicators for sharing their experiences and the Public Health Agency of Sweden for sharing quantitative data.

**REFERENCES**

1. World Health Organization. COVID-19 Response in the World Health Organization African Region, February to December 2021. Brazzaville, CG: WHO Regional Office for Africa; 2021.

2. Hansson E, Albin M, Rasmussen M, Jakobsson K. Stora skillnader i överdödlighet våren 2020 utifrån födelseland. Lakartidningen. 2020;117:28–32.

3. Yaya S, Yeboah H, Charles CH, Otu A, Labonte R. Ethnic and racial disparities in COVID-19-related deaths: counting the trees, hiding the forest. BMJ Glob Heal. 2020;5:e002913.

4. Irizar P, Pan D, Kapadia D, Bécares L, Sze S, Taylor H, et al. Ethnic inequalities in COVID-19 infection, hospitalisation, intensive care admission, and death: a global systematic review and meta-analysis of over 200 million study participants. eClinicalMedicine. 2023;57. doi.org/10.1016/j.eclinm.2023.101877

5. Marmot M, Allen J, Goldblatt P, Herd E, Morrison J. Build Back Fairer: The COVID-19 Marmot Review. The Pandemuic, Socioeconomic and Health Inequalities in England. London, UK: UCL Institute of Health Equity; 2020.

6. Rostila M, Cederström A, Wallace M, Aradhya S, Ahrne M, Juárez SP. Inequalities in COVID-19 severe morbidity and mortality by country of birth in Sweden. Nat Commun. 2023;14:4919. doi.org/10.1038/s41467-023-40568-4

7. Myndigheten för vård- och omsorgsanalys. Riktade vaccinationsinsatser: Lärdomar från regionernas arbete för en hög och jämlik vaccinationstäckning mot covid-19. Stockholm, SE: Myndigheten för vård- och omsorgsanalys; 2022.

8. Khunti K, Singh AK, Pareek M, Hanif W. Is ethnicity linked to incidence or outcomes of covid-19? BMJ. 2020;369:m1548.

9. Kluge HHP, Jakab Z, Bartovic J, D’Anna V, Severoni S. Refugee and migrant health in the COVID-19 response. Lancet. 2020;395:1237–9. doi.org/10.1016/S0140-6736(20)30791-1

10. Page KR, Venkataramani M, Beyrer C, Polk S. Undocumented U.S. Immigrants and Covid-19. N Engl J Med. 2020;382:e62. doi.org/10.1056/NEJMp2005953

11. Hitch L, Masoud D, Hobbs LA, Moujabber M, Cravero K. The vulnerability to COVID-19 of migrants in large urban areas: structural exacerbators and community-level mitigators. Eur J Public Health. 2023;33:704–16.

12. Martínez-Donate AP, Correa-Salazar C, Bakely L, González-Fagoaga JE, Asadi-Gonzalez A, Lazo M, et al. COVID-19 testing, infection, and vaccination among deported Mexican migrants: Results from a survey on the Mexico-U.S. border. Front Public Heal. 2022;10:928385.

13. Söderberg M, Cronie O, Adiels M, Rosengren A. The influence of overcrowding and socioeconomy on the spatio-temporal spread of COVID-19 - a Swedish register study. Göteborg, SE: Göteborg University; 2022.

14. Nutbeam D, Lloyd JE. Understanding and Responding to Health Literacy as a Social Determinant of Health. Annu Rev Public Health. 2021;42:159–73. doi.org/10.1146/annurev-publhealth-090419-102529

15. Wångdahl J, Lytsy P, Mårtensson L, Westerling R. Health literacy and refugees’ experiences of the health examination for asylum seekers – a Swedish cross-sectional study. BMC Public Health. 2015;15:1162. doi.org/10.1186/s12889-015-2513-8

16. Burns R, Campos-Matos I, Harron K, Aldridge RW. COVID-19 vaccination uptake for half a million non-EU migrants and refugees in England: a linked retrospective population-based cohort study. Lancet. 2022;400:S5.

17. Fernández-Sánchez H, Zahoui Z, Jones J, Marfo EA. Access, acceptability, and uptake of the COVID-19 vaccine among global migrants: A rapid review. PLoS One. 2023;18:e0287884.

18. Page KR, Genovese E, Franchi M, Cella S, Fiorini G, Tlili R, et al. COVID-19 vaccine hesitancy among undocumented migrants during the early phase of the vaccination campaign: a multicentric cross-sectional study. BMJ Open. 2022;12:e056591.

19. Lin S. COVID-19 Pandemic and Im/migrants’ Elevated Health Concerns in Canada: Vaccine Hesitancy, Anticipated Stigma, and Risk Perception of Accessing Care. J Immigr Minor Heal. 2022;24:896–908.

20. Ortega P, Martínez G, Diamond L. Language and Health Equity during COVID-19: Lessons and Opportunities. J Health Care Poor Underserved. 2020;31:1530–5.

21. Nordic Council of Ministers. Outreach and dissemination of public information to immigrants during the COVID-19 pandemic. Copenhagen, DK: Nordic Council of Ministers; 2022.

22. Hansson E, Jakobsson K. Covid-19 i trångbodda förorter och på äldreboende - samverkande strukturella faktorer? En geografisk analys av samband mellan förutsättningar för social distans och kontakter med äldre i Stockholm, Göteborg och Malmö. Göteborg, SE: Göteborg University; 2020.

23. World Health Organization. ApartTogether survey: Preliminary overview of refugees and migrants self-reported impact of COVID-19. Geneva, CH: WHO; 2020.

24. Folkhälsomyndigheten. Hur har folkhälsan påverkats av covid-19-pandemin? Samlad bedömning utifrån svensk empiri och internationell forskning under 2020. Stockholm, SE: Folkhälsomyndigheten; 2021.

25. Esaiasson P, Johansson B, Ghersetti M, Sohlberg J. Kriskommunikation och segregation i en pandemi: Hur boende i utsatta områden informerade sig om coronaviruset våren 2020. Göteborg, SE: Göteborg University; 2020.

26. Cai D-Y. A concept analysis of cultural competence. Int J Nurs Sci. 2016;3:268–73.

27. Metzl JM, Hansen H. Structural competency: Theorizing a new medical engagement with stigma and inequality. Soc Sci Med. 2014;103:126–33.

28. Ahearn LM. Language and Agency. Annu Rev Anthropol. 2001;30:109–37. doi.org//10.1146/annurev.anthro.30.1.109

29. Bresnahan M, Zhuang J. Culturally safe healthcare: changing the lens from provider control to patient agency. J Commun Healthc. 2024;17:244–53

30. Statistiska centralbyrån. Statistikdatabasen. Örebro, SE: SCB; 2022.

31. Björkman A, Gisslén M, Gullberg M, Ludvigsson J. The Swedish COVID-19 approach: a scientific dialogue on mitigation policies. Front Public Heal. 2023;11. doi.org/10.3389/fpubh.2023.1206732

32. Smittskydd Stockholm. Epidemiberedskapsplan, Region Stockholm. Stockholm, SE: Smittskydd Stockholm; 2019.

33. Bäärnhielm S, Al-Ammari B, Hussein H. Erfarenheter från Region Stockholms telefonlinje om covid-19 på olika språk: Interkulturell kommunikation i samarbete mellan vård och civilsamhälle. Soc Tidskr. 2021;124–30.

34. United States Centers for Disease Control and Prevention. Crisis & Emergency Risk Communication (CERC) Manual. Atlanta, GA: CDC; 2018.

35. Vaughan E, Tinker T. Effective Health Risk Communication About Pandemic Influenza for Vulnerable Populations. Am J Public Health. 2009;99:S324–32. doi.org/10.2105/AJPH.2009.162537

36. Braun V, Clarke V. Thematic Analaysis: A Practical Guide. Thousand Oaks, CA: SAGE Publications; 2021.

37. Kvale S. InterViews: An introduction to qualitative research interviewing. InterViews: An Introduction to Qualitative Researh Interviewing. Thousand Oaks, CA: Sage Publications; 1994.

38. Desmarais C, Roy M, Nguyen MT, Venkatesh V, Rousseau C. The unsanitary other and racism during the pandemic: analysis of purity discourses on social media in India, France and United States of America during the COVID-19 pandemic. Anthropol Med. 2023;30:31–47. doi.org/10.1080/13648470.2023.2180259

39. Abdi N. “Somalierna fick skulden för sin egen död”: En kvalitativ studie av hur somalier i Järvområdets upplevt medierapporteringen under Coronapandemin. Uppsala, SE: Uppsala University; 2021

40. Kasstan B, Mounier-Jack S, Gaskell KM, Eggo RM, Marks M, Chantler T. “We’ve all got the virus inside us now”: Disaggregating public health relations and responsibilities for health protection in pandemic London. Soc Sci Med. 2022;309:115237

41. Peters MDJ. Addressing vaccine hesitancy and resistance for COVID-19 vaccines. Int J Nurs Stud. 2022;131:104241

42. Dutta-Bergman MJ. Theory and Practice in Health Communication Campaigns: A Critical Interrogation. Health Commun. 2005;18:103–22. doi.org/10.1207/s15327027hc1802_1

43. Hanlon P, Carlisle S, Hannah M, Reilly D, Lyon A. Making the case for a “fifth wave” in public health. Public Health. 2011;125:30–6.

44. Brewis A, Wutich A. Lazy, Crazy, and Disgusting: Stigma and the Undoing of Global Health. Baltimore, MD: Johns Hopkins University Press; 2019.

45. Short SE, Mollborn S. Social Determinants and Health Behaviors: Conceptual Frames and Empirical Advances. Curr Opin Psychol. 2015;5:78–84.

46. Baum F, Fisher M. Why behavioural health promotion endures despite its failure to reduce health inequities. Sociol Health Illn. 2014;36:213–25. doi.org/10.1111/1467-9566.12112

47. Zoller HM, Kline KN. Theoretical Contributions of Interpretive and Critical Research in Health Communication. Ann Int Commun Assoc. 2008;32:89–135. doi.org/10.1080/23808985.2008.11679076

48. Miklavcic A, LeBlanc MN. Cultural Brokers, Clinical Applied Ethnography, and Cultural Mediation. In: Kirmayer LJ, Guzder J, Rousseau C, editors. Cultural Consultation: Encountering the Other in Mental Health Care. New York, NY: Springer Nature; 2014. p. 115–37.

49. Monson K, Oluyinka M, Negro D, Hughes N, Maydan D, Iqbal S, et al. Congregational COVID-19 Conversations: Utilization of Medical-Religious Partnerships During the SARS-CoV-2 Pandemic. J Relig Health. 2021;60:2353–61.

50. Wijesinghe MSD, Ariyaratne VS, Gunawardana BMI, Rajapaksha RMNU, Weerasinghe WMPC, Gomez P, et al. Role of religious leaders in covid-19 prevention: A community-level prevention model in Sri Lanka. J Relig Health. 2022;61:687–702.

51. Bruce MA. COVID-19 and African American Religious Institutions. Ethn Dis. 2020;30(3):425–8.

52. Kasstan B. Vaccines and vitriol: an anthropological commentary on vaccine hesitancy, decision-making and interventionism among religious minorities. Anthropol Med. 2021;28:411–9. doi.org/10.1080/13648470.2020.1825618

53. Lagadec P. The Unknown Territory of Mega-Crisis: In Search of Conceptual and Strategic Breakthroughs. In: Helsloot I, Boin A, Jacobs B, Comfort LK, editors. Mega-Crises: Understanding the Prospects, Nature, Characteristics and Effects of Cataclysmic Events. Springfield, IL: Charles C Thomas Publisher; 2012. p. 12–24.

54. Yalom ID. Existential Psychotherapy. New York, NY: Basic Books; 1980.

55. Ahmed N, Barnett P, Greenburgh A, Pemovska T, Stefanidou T, Lyons N, et al. Mental health in Europe during the COVID-19 pandemic: a systematic review. Lancet Psychiatry. 2023;10:537–56. doi.org/10.1016/S2215-0366(23)00113-X

56. Santabárbara J, Lasheras I, Lipnicki DM, Bueno-Notivol J, Pérez-Moreno M, López-Antón R, et al. Prevalence of anxiety in the COVID-19 pandemic: An updated meta-analysis of community-based studies. Prog Neuro-Psychopharmacology Biol Psychiatry . 2021;109:110207

57. Fahriani M, Ilmawan M, Fajar JK, Maliga HA, Frediansyah A, Masyeni S, et al. Persistence of long COVID symptoms in COVID-19 survivors worldwide and its potential pathogenesis - a systematic review and meta-analysis. Narra J. 2021;1:e36.

58. Kitchingman TA, Wilson CJ, Caputi P, Wilson I, Woodward A. Telephone Crisis Support Workers’ Psychological Distress and Impairment. Crisis. 017;39:13–26. doi.org/10.1027/0227-5910/a000454

59. Hamdan A, Eastaugh J, Snygg J, Naidu J, Alhaj I. Coping strategies used by healthcare professionals during COVID-19 pandemic in Dubai: A descriptive cross-sectional study. Narra X. 2023;1.

60. Mheidly N, Fares J. Leveraging media and health communication strategies to overcome the COVID-19 infodemic. J Public Health Policy. 2020;41:410–20. doi.org/10.1057/s41271-020-00247-w

61. Chong YY, Cheng HY, Chan HYL, Chien WT, Wong SYS. COVID-19 pandemic, infodemic and the role of eHealth literacy. Int J Nurs Stud. 2020;108:103644.

62. van der Linden S, Roozenbeek J, Compton J. Inoculating Against Fake News About COVID-19. Front Psychol. 2020;11:566790.

63. Sveriges Kommuner och Regioner. Att lära av en kris: Kommuners och regioners lärdomar från covid-19-pandemin. Stockholm, SE: SKR; 2023.

64. Altmejd A, Östergren O, Björkegren E, Persson T. Inequality and COVID-19 in Sweden: Relative risks of nine bad life events, by four social gradients, in pandemic vs. prepandemic years. Proc Natl Acad Sci. 2023;120:e2303640120. doi.org/10.1073/pnas.2303640120

65. Busetto L, Wick W, Gumbinger C. How to use and assess qualitative research methods. Neurol Res Pract. 2020;2:14. doi.org/10.1186/s42466-020-00059-z

66. Graneheim UH, Lindgren B-M, Lundman B. Methodological challenges in qualitative content analysis: A discussion paper. Nurse Educ Today. 2017;56:29–34.
