# Supplementary material for: A multilingual telephone service for crisis communication with migrant groups: Swedish experiences of responding to the COVID-19 pandemic
Source: BMC Public Health. 2026 Feb 4;26:723. doi: 10.1186/s12889-026-26413-5 (PMC12931007; doi:10.1186/s12889-026-26413-5)
Supplement: Supplementary file 3 — Supplementary Material 3. [file 12889_2026_26413_MOESM3_ESM.docx]

Highlights

- Experiences from a multilingual telephone service for the dissemination of public health information about COVID-19 and vaccination to migrants during the pandemic point to the value of establishing trust through crisis communication that includes the opportunity for dialogue with health professionals in a culturally safe mode using one's native language.
- For future health crises, our findings emphasize the importance of establishing a pre-existing crisis communication strategy targeting vulnerable groups as a part of a comprehensive pandemic plan when the need emerges.
