# Supplementary material for: A multilingual telephone service for crisis communication with migrant groups: Swedish experiences of responding to the COVID-19 pandemic
Source: BMC Public Health. 2026 Feb 4;26:723. doi: 10.1186/s12889-026-26413-5 (PMC12931007; doi:10.1186/s12889-026-26413-5)
Supplement: Supplementary file 4 — Supplementary Material 4. [file 12889_2026_26413_MOESM4_ESM.docx]

**Title: A multilingual telephone service for crisis communication with migrant groups: Swedish experiences of responding to the COVID-19 pandemic**

**Authors:** Sofie Bäärnhielm, Baidar Al-Ammari, Önver Cetrez, Soorej Jose Puthoopparambil, Mattias Strand

**Keywords:**

COVID-19, communication, language, culture, health crisis, pandemic plan

**Corresponding author:** Sofie Bäärnhielm

Email: [sofie.baarnhielm@ki.se](mailto:sofie.baarnhielm@ki.se)

Address:

Transcultural Center, Region Stockholm

Solnavägen 1E floor 7, 113 65, Stockholm, Sweden

**ABSTRACT**

**Background:** Migrants living in socioeconomically disadvantaged neighborhoods in Sweden were overrepresented among the infected and deceased in coronavirus disease 2019 (COVID-19) and vaccination coverage was substantially lower, despite being free of charge. The overarching aim of this study was to analyze the experiences of operating a multilingual telephone service for public health crisis communication targeting migrant communities in Sweden during COVID-19. A secondary objectives was to identify specific opportunities and challenges in delivering culturally appropriate health information during a pandemic crisis.

**Methods:** A qualitative design based on in-depth interviews with 12 health communicators staffing the telephone service was used. Additional quantitative descriptive data on the use of the telephone service are provided for context.

**Results:** The quantitative data revealed that relatively few callers requested basic information about the virus or asked about topics such as where to turn in case of illness. The most common topic was testing for current infection. The thematic analysis identified seven major themes: The rationale behind a multilingual telephone service; the convergence of language, culture, and professional competence in trust building; dialogical dissemination of knowledge; cooperation with other actors and organizations; responding to misinformation and myths; managing emotions, existential concerns, and mental distress; and lessons for future health crisis response.

**Conclusions:** For dissemination of information about COVID-19 and vaccination to migrants during the pandemic, experiences from the multilingual telephone service point to the value of communication that includes the possibility of dialogue with health professionals in a culturally safe mode using one's native language. For future health crises, our findings emphasize the importance of having a communication strategy targeting vulnerable groups in place as a part of a comprehensive pandemic plan when the need emerges.

**Trial registration:** The study protocol has been preregistered on the Open Science Framework (osf.io/rt47j)

**BACKGROUND**

“No-one is safe until we are all safe”— the words of World Health Organization (WHO) Director-General and the mantra of the WHO during the coronavirus disease 2019 (COVID-19) pandemic—point to the global importance of ensuring that all groups in a society are being protected from the virus [1]. A crucial component in reaching this goal is accurate and effective public health crisis communication about the virus and the recommended precautions. For multicultural societies, this includes the challenge of reaching and establishing a dialogue with diverse sociocultural, linguistic, and religious groups. The COVID-19 pandemic has revealed problems at individual, group, and structural levels and highlighted the importance of inclusive health communication strategies that reflect the needs and contexts of various population subgroups in a society.

In Sweden, as elsewhere, migrant groups living in socioeconomically disadvantaged neighborhoods were overrepresented among the infected and deceased [2–5]. For the entire duration of the pandemic in Sweden, a substantially higher risk of COVID-19 intensive care unit admission was seen among migrants from Africa, Middle-East, Asia, South America, and other European countries compared to Swedish-born individuals [6]. Similar patterns were seen for mortality in COVID-19 among groups with different geographical origin. Moreover, despite the fact that COVID-19 vaccination was free of charge and implemented with the ambition of reaching the whole Swedish population above 15 years of age, vaccination coverage in Sweden was substantially lower in the same socioeconomically disadvantaged multicultural areas that were most severally hit in the first pandemic wave [7].

These findings in the Swedish context mirror those observed on a global scale. Racial and ethnic disparities in COVID-19-related morbidity and mortality, not least affecting refugees and asylum seekers, were reported from multiple countries early on in the pandemic [8–10]. In retrospect, it is evident that the COVID-19 pandemic disproportionately affected migrant communities worldwide, largely due to structural inequality, residential segregation, and barriers to healthcare [4,11,12]. Individuals with a migration background tend to more often be employed in the service sector, where working from home is seldom an option, or in healthcare and care for the elderly, where the risk of acquiring and/or transmitting infections may be higher [13]. Cramped housing accommodation and a tendency for more socializing across generations might also have contributed to higher burden of disease [11,13]. Moreover, migrants who are new to a country may find it difficult to navigate the healthcare system, which becomes an obstacle in comprehending medical information [14,15]. Importantly, migrant populations typically had relatively low vaccine uptake once COVID-vaccines became available, the reasons for which included limited access, stigma, and vaccine hesitancy [12,16–19].

Information from public authorities that is poorly adapted to the particular needs of migrant groups has been suggested as another important contributing cause behind the observed patterns [20,21]. Official recommendations need to be adapted to real-life conditions in order to be seen as understandable, relevant, and trustable. For example, working from home or avoiding public transportation is unfeasible for certain groups in society, such as many refugees and migrants, for whom it may be more useful with hands-on guidelines on how to best handle cramped living conditions [22,23]. Population groups that perceive official recommendations as unrealistic or irrelevant to their particular situation risk feeling less valued as citizens, becoming alienated, and putting less trust in government agencies [24].

Furthermore, knowledge of the local language may be poor, especially among elderly migrants and those who have newly arrived in a country [15]. Some may also have to rely on verbal rather than written information due to illiteracy. As reported on a global level as well as in Sweden, a significant proportion of refugees and migrants relied on news from their country of birth as the main source of information about COVID-19 [23,25]. This may have had a negative impact on the knowledge of local recommendations and restrictions.

Importantly, several of these mechanisms may interact in giving rise to feelings of exclusion, alienation, and lack of trust. In the public health work of government agencies, ‘cultural competence’ as well as ‘structural competence’ are therefore necessary components of any successful intervention. The concept of cultural competence can be defined as healthcare professionals’ gradually developed capacity to provide safe and high-quality healthcare to patients of different cultural backgrounds, with defining attributes such as cultural awareness, cultural sensitivity, cultural knowledge, cultural skills, and an emphasis on *becoming* rather than *being* culturally competent [26]. Structural competence in healthcare, in turn, refers to the ability to recognize how health is affected by broad social, political, and economic structures, including healthcare systems, food availability, local infrastructure, zoning laws, etc. [27]. Importantly, cultural and structural competence are vital components in identifying barriers to patient *agency*—which can be defined as a socio-culturally mediated capacity to act [28]—and self-determination in health behaviors and healthcare, especially so for marginalized groups [29].

Two factors contributed in making intercultural health crisis communication a particularly pressing issue in Sweden during the early stages of the COVID-19 pandemic. First, Sweden has a relatively large migrant population compared to many other European countries. Foreign-born persons currently make up 20.4% of the Swedish population; when including persons born in Sweden to two foreign-born parents, the number is 26.8% [30]. Second, contrary to most other countries, Sweden did not enforce a strict lockdown policy during the pandemic. Instead, Swedish authorities opted to rely primarily on voluntary public health measures for mitigating the spread of COVID-19, on the grounds that this strategy would be more sustainable [31]. The Swedish pandemic approach proved to be a controversial choice that required well-measured communication efforts from governmental agencies, not least towards groups in society that tend to rely less on ‘mainstream media’ and more on foreign news outlets and social media [25]. Notably, at the outset of the COVID-19 pandemic, the newly updated epidemic preparedness plan of Region Stockholm [32] did not include any measures to reach various local population groups.

**A multilingual telephone service**

To reach non-Swedish-speaking migrant communities in the greater area of Stockholm, the Transcultural Center—the public resource center for migrant health in Region Stockholm—took the initiative to launch a regional multilingual telephone service for questions concerning COVID-19 and vaccination in April 2020 [33]. In June 2020, this telephone service was nationalized in order to serve the whole country, in cooperation with the Public Health Agency of Sweden (PHAS). It operated until April 2022. The telephone service did not offer individual-level medical advice; instead, its primary purpose was to answer questions and queries of a more general character about COVID-19 and vaccination in minority languages. The service was open between 9 AM and 3 PM on weekdays. Callers could choose between a number of minority languages: Arabic, Amharic, Dari/Farsi, English, Russian, Serbo-Croatian, Somali, Spanish (available intermittently), and Tigrinya.

The national telephone service was staffed by multilingual health professionals, most of whom were so-called health communicators, who were employed by Region Stockholm or Region Östergötland (a region in southeast Sweden), although they answered calls from the whole country. The HCs are individuals with a migration background of their own. They all have an education in medicine or public health; a typical background of a HC may be someone who worked as a nurse or a medical doctor in their country of origin, but who has not yet been able to validate their diploma to work clinically in Sweden. Before the pandemic, the HCs worked in local contexts within a public health intervention aimed to increase personal health literacy and facilitate help seeking among migrants. This work involved meeting newly arrived refugees and migrants living under socially precarious and stressful conditions. In their work with the multilingual telephone service, the HCs worked from home with weekly digital team meetings and advisory meetings with the PHAS and Smittskydd Stockholm (the regional infection control authority), addressing up-to-date recommendations and offering support on how to answer new or difficult questions.

The telephone service was funded by Region Stockholm, Region Östergötland, and the PHAS. It was promoted through established governmental and regional information outlets (such as official websites, advertisements in newspapers and public transportation, etc.). There were also additional promotional efforts in close collaboration with various partner networks, including civic society organizations who regularly meet migrant groups.

**Theoretical framework and aims**

This study builds on the theoretical framework for health crisis communication outlined by the United States Centers for Disease Control and Prevention (CDC) and others, emphasizing the phased, situation-specific, and culturally sensitive nature of effective outreach and community engagement with vulnerable populations [34,35]. This framework is centered around the necessity of building *trust* (i.e., individual- and community-level confidence in the competence, fairness, transparency, and accountability of risk management leadership) in times of crisis. This capacity is, in turn, influenced by crisis psychology—acknowledging that people tend to process and act on information differently under stress than they would in non-crisis contexts [34]—and can be strengthened by iterative communication strategies that actively engage vulnerable populations and acknowledge their cultural orientations, priorities, and lived realities [35]. Notably, ‘hotline’ telephone services, such as the one described here, are listed by the CDC as a typical examples of a high-level community engagement activity.

The overall aim of this study was to analyze the experiences of operating a multilingual telephone service for COVID-19 public health communication targeting migrant communities in Sweden, identifying key factors that contributed to successful implementation and barriers encountered. Secondary objectives were to examine experiences of different communication strategies used to convey COVID-19 and vaccination information to lay persons from diverse linguistic backgrounds; and to identify specific opportunities and challenges in delivering culturally appropriate health information during a pandemic crisis. In the longer term, we hoped to contribute to the development of evidence-based recommendations for strengthening intercultural health communication capacity in future public health emergencies.

**METHODS**

**Study design**

For this study, a qualitative research design based on several individual in-depth interviews and one focus group interview with health communicators (HCs) staffing the telephone service was employed. Moreover, in order to provide some additional context, quantitative descriptive data from the telephone service are presented.

**Data collection and analysis**

Quantitative data

Descriptive data on the multilingual COVID-19 telephone service were collected and documented in survey-format by the PHAS from June 1, 2020 until April 30, 2022. These survey data were entered in real-time by the health professionals staffing the telephone service and included the regional location of the callers, the preferred language used, the specific topics raised during the calls, and whether the callers were referred to the government-run national healthcare telephone service “1177”, operated by nurses and other healthcare professionals. An optional free-text entry for further information was also available. No external validation of the staff’s data entry was performed. The data were accessed with permission from the PHAS; however, any information that could potentially compromise confidentiality was deleted by the PHAS. A descriptive statistical analysis was performed utilizing IBM® SPSS® Statistics version 28. Missing data were excluded from the analysis.

Qualitative data

An interview guide was created, covering the following areas: Experiences of organizing the telephone service; the type of questions received; how questions changed over time; experiences of answering questions; perception of the importance of language; and the perception of key factors to establishing trust and report with callers (see Appendix 1). These areas were chosen based on the identification on three domains of language barriers, cultural issues, and structural issues outlined above as well as on the findings in the quantitative component. To ensure maximum relevance, the interview guide was designed with active input from one of the HCs who had staffed the telephone line and who participated as a researcher in the project. The included items were phrased so as to assure informants that their individual performances were not being evaluated and that the aim of the interviews were to identify overarching themes that might guide similar crisis response interventions in the future.

The sole criterion for inclusion in the qualitative interviews was that informants should have staffed the multilingual telephone service during the pandemic. No explicit exclusion criteria were employed. Individual in-depth face-to-face interviews were conducted with six of the seven HCs in Region Stockholm that staffed the telephone service; the seventh HC participated as a researcher in the project and was not interviewed. These interviews lasted between 30 and 90 minutes. An in-person focus group interview lasting 90 minutes was conducted with the six HCs from Region Östergötland. All interviews were performed in Swedish. Since the qualitative interviews included all of the HCs staffing the telephone service in Region Stockholm and Region Östergötland (except for the HC participating as researcher), an almost full coverage was achieved. In order to mitigate any informant bias (such as a potential tendency to exaggerate the benefits and success of the telephone service), the interviews explicitly focused on areas of potential improvement for future health crises. An overall impression is that the informants did not express any concern in voicing potentially ‘uncomfortable’ opinions and that the interview data contained a vast array of potential areas of improvement.

All interviews were digitally recorded and transcribed verbatim. The transcripts were anonymized by omitting or changing details that could risk identifying persons who had contacted the telephone service.

A thematic analysis framework was used in the analysis of the interview data [36]. From an epistemological perspective, this framework reflects a view of knowledge and meaning as created through social interactions and experiences that are necessarily interpreted through the lens of cultural, contextual, and individual-level factors. Our analytical approach was explorative and inductive and thus not based on pre-established theoretical conceptions [37]. First, all authors familiarized themselves with the interview data. Two authors (BAA and SB) then coded the data together, identifying meaning units and developing preliminary codes, categories, and themes. These were discussed and revised in an iterative process involving both coders, until a preliminary interpretation of the content and categorization of the meaning units were agreed on. This interpretation was then discussed with all authors, who participated in a final revision of the themes and subthemes.

**Ethics and preregistration**

This study was conducted in accordance with the ethical standards of the Helsinki Declaration of 1975, as revised in 2008. The study was approved by the Swedish Ethical Review Authority (No. 2022-01637-01). Written consent was obtained from all participants. To protect the integrity and safety of the HCs and to avoid potential of identifying the participants, we have chosen not to provide any personal information to the quotes. The study protocol has been preregistered on the Open Science Framework (osf.io/rt47j).

**RESULTS**

**Quantitative data**

There were, in total, 9 414 calls to the telephone service. Of these calls, 4 150 were registered and documented in survey-format by the PHAS. The most commonly requested languages were Arabic (33.8%), English (29.8%), Persian/Dari (14.9%), Tigrinya (9.6%), and Russian (5.6%). Few callers requested information in Somali (2.5%), and even fewer in Amharic (1.1%), Serbo-Croatian (<1%), and Spanish (<1%).

A large majority of callers brought up only one or two topics (74.0% and 20.1%, respectively). The single most common topic was polymerase chain reaction (PCR) testing for *current* COVID-19 infection, raised in 39.6% of all calls. Fewer calls concerned the topic of antibody testing for *past* COVID-19 infection (3.6% of all calls). Another very common topic was how to acquire a travel certificate after a PCR test (26.0% of all calls). The distribution among languages was fairly equal for these topics.

In 14.7% of all calls, the question about when and how to get vaccinated was raised. Notably, of all calls in Tigrinya, 33.0% (132 calls) concerned this topic. Calls about vaccine safety and side effects were few (2.6% and 2.3%, respectively). Here too, calls in Tigrinya stand out, having raised this topic in 12.0% and 7.0% of all calls, respectively. Only 5.3% of all calls concerned the topic of *why* to get vaccinated, with no large differences between languages. Also noteworthy, calls about travel certificates after vaccination were much less common (2.5% of all calls) than calls about travel certificates after PCR testing.

Of all calls, only 5.0% brought up the topic of where to turn in case of COVID-19 illness. However, this topic was raised in 17.4% of all calls in Somali, in 9.9% of all calls in Russian, and 9.4% of all calls in Arabic. Overall, few callers requested basic information about what the corona virus is (0.9%), how the virus is spread (1.2%), at-risk groups (1.4%), how to avoid becoming infected (1.3%), how to avoid infecting others (2.1%), or how to access more information about the corona virus (3.7%).

In 16.0% of all calls, other topics than those listed above were raised. The free-text entries provided in the PHAS data sheet indicate that some of these calls concerned practical issues, such as how to book a vaccination appointment without electronic identification, how to get tested without having a national social security number, vaccination appointments for asylum seekers, etc. Having had a first vaccine dose in a foreign country was also raised in a number of calls. Other topics concerned quarantine, participation in outdoor sports, and concern about public facilities not following recommended guidelines.

In total, 56.6% of all calls resulted in a referral to the national healthcare telephone service “1177”. Notably, 87.5% of Somali-speaking callers were referred to “1177”.

With the quantitative results indicating the migratory background of the callers and the common questions asked, we now move on to the qualitative results that provide an in-depth understanding on how the HCs experienced the calls, the challenges they faced and how they addressed them.

**Qualitative data**

The thematic analysis identified seven major themes: 1) The rationale behind a multilingual telephone service; 2) the convergence of language, culture, and professional competence in trust building; 3) dialogical dissemination of knowledge; 4) cooperation with other actors and organizations; 5) responding to misinformation and myths; 6) managing emotions, existential concerns, and mental distress; and 7) lessons for future health crisis response.

Theme 1: The rationale behind a multilingual telephone service

This theme deals with the HCs’ perception of the need for and rationale behind a multilingual telephone service, informed by their experience in setting it up and adapting it over time. At the beginning of the pandemic in particular, the HCs experienced a great demand for information in their native languages. Infection rates were high in migrant-dense areas, yet information from the government in multiple languages was lacking. Once the multilingual telephone service was introduced, the HCs noticed that many callers had some limited knowledge of Swedish, but not on the level required for understanding the somewhat technical and complex information from the authorities. The HCs also highlighted the importance of verbal dialogue through the telephone service, as literacy barriers affected some migrants.

Importantly, the type of questions the HCs got changed as the pandemic progressed, reinforcing the rationale for a responsive telephone service that could address evolving concerns. Early on, they mostly received questions about virus transmission, protection against infection, symptoms of COVID-19, testing, and how to access medical emergency treatment. Later, as vaccines against COVID-19 became available, more questions concerned vaccination. This ‘moving target’ character of the pandemic sometimes proved challenging for the HCs, as established health communication protocols were insufficient for an evolving crisis in which information became outdated within days.

However, callers did not only have straightforward questions about public health aspects of COVID-19. Many questions focused on personal social and economic consequences of the pandemic—issues that were more challenging for the HCs to address since they fell outside of their area of expertise. Still, this emphasizes the rationale for an interactive service that could at least acknowledge these broader concerns, even when direct solutions were not available. Moreover, many callers posed questions related to their own health issues and current symptoms. The HCs had neither the mandate nor the expertise to answer these types of medical questions. Instead, they were instructed to refer callers to the official Swedish healthcare telephone service, “1177”, staffed by nurses and other healthcare professionals. Even so, the fact that the HCs could and should not answer medical questions occasionally caused some frustration among callers.

Theme 2: The convergence of language, culture, and professional competence in trust building

The central role of establishing trust with callers was emphasized by the HCs. Their impression was that the combination of a shared language, knowledge of different cultures and contexts, and the explicit status of the HCs as representatives of the Swedish healthcare system contributed in trust formation. Building trust was an iterative process during the conversation with callers:

*“Usually, you started out with something—with a question—and then when [the callers] felt reassured they would ask, ‘okay, can I ask something else, it may not be relevant but I think you are the right person to ask these questions to’.”*

According to the HCs, the opportunity to communicate in one's native language proved essential for accurate comprehension of health information and government directives. This was particularly important given that callers often needed to ask clarifying questions about health issues that government agencies might assume were commonly understood (such as the difference between viruses and bacteria or how vaccines provides protection).

Furthermore, the HCs' cultural backgrounds and insider perspectives on callers' sociocultural contexts were viewed as crucial for tailoring responses appropriately and, consequently, building trust. For example, one HC contrasted generic health advice such as "drink hot beverages" with culturally informed recommendations that draw on specific cultural practices, such as suggesting green tea to Afghan callers who already have this as part of their daily routine. By utilizing emic insight and lived experiences as migrants to Sweden, the HCs were thus able to transform impersonal medical advice into personalized guidance that connects with callers' cultural contexts, thereby facilitating trust and relatability. The HCs also emphasized that many callers came from countries and communities where trust in authorities and the healthcare system is generally low, not seldom for good reasons. Here too, cultural sensitivity became essential, as HCs had to demonstrate understanding of callers' backgrounds and validate their concerns while gradually building trust.

A final important factor in building trust through credibility was the professional competence of the HCs and their explicit affiliation with the Swedish healthcare system. The HCs described how, by embodying both professional knowledge and systemic legitimacy, they positioned themselves as trustworthy intermediaries between patients and the healthcare system.

Theme 3: Dialogical dissemination of knowledge

This theme refers to the HCs' perception that they could contribute knowledge that callers not only received and understood but also ultimately applied to change their behavior. For successful dissemination of scientific evidence related to the COVID-19 pandemic, the HCs emphasized that callers needed opportunities to ask questions, share their perspectives, and engage in dialogue about different viewpoints, instead of receiving only one-way information. The HCs would often receive feedback from callers confirming that they had modified their views on COVID-19 and vaccination , with real-world consequences for their behavior:

*“This caller had read a lot about [vaccination against COVID-19] on social media: [exclaiming] 'This is how it is, you provide the wrong information.' [But] finally, he accepted. He called me twice. But he accepted [and told me]: ‘Yes, I will get vaccinated’.”*

When the HCs perceived that they succeeded in disseminating knowledge, they gained the impression that this information was also being shared with others in the community. Callers often mentioned that they intended to pass it on to family, friends and acquaintances. When callers expressed clear skepticism about vaccination, it was important that the HCs allowed them sufficient time to talk. The HCs described how even those who were initially quite firm in their beliefs could eventually change their minds. Some of those who contacted the telephone service ended up calling several times. The possibility to call repeatedly was considered important for conveying new messages about COVID-19 and vaccination. It gave callers the opportunity to reflect, consult multiple sources of information, and return with new questions.

Notably, it was sometimes important for the HCs to respond credibly to personal questions and to act as role models, further underscoring the relational dynamics of the telephone service. This was particularly relevant at the start of the vaccination program, when they were asked to explain why they themselves had not been vaccinated:

*“In the beginning it was a bit difficult, when the vaccine arrived and we hadn't been offered the first dose. ‘Oh, you're not a good role model, you have to get vaccinated first’. Questions like that. One time I was asked, ‘Have you been vaccinated?’. They started vaccinating the older ones and I was waiting for my age [group to be offered the vaccine]. ‘Do you think you will get vaccinated?’ ‘You have to get vaccinated before you talk [to others] about vaccines!’”*

One challenge that the HCs identified as negatively affecting knowledge dissemination was that the marked differences between the Swedish pandemic strategy and those of other countries. Callers were aware of the guidelines being implemented in their countries of origin and the HCs needed to take time to discuss these differences. Callers would also often express confusion related to the Swedish approach. One HC put it this way:

*“Sweden had slightly different guidelines compared to other countries, and if people watch [television] channels from their home countries and they see these very strict rules, with face masks, lockdown, no one going to school, then there’s a lot of confusion among the callers about who is right and who is wrong.”*

When this topic arose, the HCs did not try to persuade the callers of the Swedish pandemic strategy; instead, they simply explained the guidelines and the official rationale behind them. They also urged callers to consult government sources for relevant information. While this might seem at odds with the general aim of dialogue-based interaction, it represented a necessary balance between fostering open conversation and ensuring that callers received reliable, up-to-date information.

In their interaction with migrants, the HCs also emphasized behavioral change as a way of showing consideration for others in the community. One HC described engaging with callers who were hesitant to get vaccinated because they believed their own immune system was sufficient, illustrating how the telephone service facilitated dialogue that addressed not only individual decisions but also their potential consequences for the broader population:

*“But we told them about the importance of taking responsibility for others too. You get vaccinated for your own sake, but also to protect your loved ones and everyone else in the community.”*

In sum, the opportunity for dialogue, as opposed to one-way, top-down communication, was emphasized as a main strength in enabling successful dissemination of knowledge.

Theme 4: Cooperation with other actors and organizations

Effective collaboration with other organizations in promoting the telephone service was stressed by the HCs. Extensive efforts were made to reach community-level organizations likely to have contact with the telephone service’s target groups. Cooperation with civil society not only for raising public awareness of the telephone service, but also to deliver a consistent message about COVID-19 and vaccination. Reaching out to and engaging religious communities was seen as particularly helpful. Religious leaders and the messages they conveyed were perceived as having a significant influence on people's attitudes toward COVID-19 and vaccination. The HCs used their out-of-office hours and their networks to share information on COVID-19, vaccination, and the telephone service:

*“I have spread the information in churches and left brochures in [local] shops. I usually do it in my spare time, leaving brochures with health information and information about the telephone service. In this way, I have also been in contact with the housing agency to disseminate information.”*

Other key partners for the HCs included schools teaching Swedish for migrants, educational associations, and housing companies.

Theme 5: Responding to misinformation and myths

Working with the telephone service sometimes involved responding to misinformation and widespread myths in society concerning the pandemic’s origin, virus transmission, and the vaccines. Although many callers were clearly influenced by such misinformation, they were seldom considered completely inflexible in their views. Callers were typically perceived to possess at least some curiosity, which enabled meaningful interaction. Some appeared to call to learn arguments they could use in discussions with others and to build the confidence to get vaccinated themselves:

*“[T]here were those who had some doubts and wanted some kind of reassuring confirmation that it’s not dangerous and that it’s the best remedy and that the myths that flourish or are being spread aren’t true.”*

Nevertheless, the HCs faced clear challenges in addressing misinformation and myths about COVID-19 and vaccination. Callers often referred to many different types of sources of COVID-19-related information, and sometimes to an explicit lack of information. Common sources included friends and family, religious communities and leaders, and social media. As noted earlier, many specifically referred to information from their countries of origin, which was not necessarily relevant to their local context. Importantly, the HCs noted that fear was a clear motivation for callers engaging with misinformation. Accordingly—as discussed in more detail below—being open to addressing people’s fears was crucial for questioning myths and misconceptions:

*“Many times it’s not easy, because they fear for their lives and are very convinced. But the fact that they call is a positive thing. If you're absolutely determined and don't want to be vaccinated, for example, then you wouldn't call.”*

One approach that appeared helpful in addressing misinformation and myths was to frame arguments in terms of current scientific understanding, while maintaining a dialogical stance that acknowledged callers’ concerns. For instance, a common concern among callers was that the vaccine had been developed quickly and might have unknown long-term effects. In these conversations, the HCs emphasized the importance of critically considering sources of information and encouraged callers to consider the wider body of evidence.

In sum, misinformation was abundant during the COVID-19 pandemic, but the HCs were generally able to find ways to counteract myths and misconceptions through active dialogue and by adhering to established evidence.

Theme 6: Managing emotions, existential concerns, and mental distress

This theme reflects how questions about COVID-19 were often expressed in narratives marked by worry, fear, loneliness, social hardship, personal difficulties, and loss. The HCs gave many examples of conversations with callers who were fearful about virus transmission and worried about their own health, often intertwined with broader worries about social disruptions, economic consequences of the pandemic, and the well-being of friends and relatives in their countries of origin. It was often challenging for the HCs to deal with the strong emotional reactions they encountered, highlighting the emotional labor involved in responding to anxiety, distress, and uncertainty during the pandemic. For example, one HC described how reports of COVID-19 deaths in Iran led to heightened anxiety and distress among members of the Iranian diaspora, who feared for the safety of their loved ones and felt a sense of helplessness due to geographical separation. Notably, several calls to the telephone service revealed broader existential concerns, including loneliness and isolation, particularly during the early stages of the pandemic when regular health-related support systems were disrupted:

*“[T]hey were feeling very bad and isolated. In our culture, when you are sick, people come and visit you. But in this case, during the pandemic, everyone was afraid to see each other and therefore the loneliness affected them a lot, they felt bad mentally. Many people asked us where they can turn.”*

Fear among callers could also make it more challenging to effectively convey accurate information and guidance. Sometimes callers did not have a specific question but primarily needed someone to share their worries with. In such cases, the HCs would simply listen and provide support:

“*Most of the time, it was about them wanting to talk. Because [here] they actually had the chance to talk. Sometimes you had to say that there were other calls waiting, but they just wanted to keep talking. It was difficult—the night is long when one has COVID […]. They get stressed and it's hard for us to talk for a long time, but I sometimes used to give them [more] time and talk to them [at length].”*

All HCs had prior experiences of working with refugees and migrants in difficult situations involving mental distress. Their approach was to guide people to appropriate mental health resources, without acting as mental health professionals or counselors themselves. Here, the HCs navigated a fine line between providing guidance and getting too personally involved. The HCs received support from the PHAS and Smittskydd Stockholm through regular meetings, educational sessions, and rapid assistance regarding new questions. However, this support focused primarily on issues related to the COVID-19 virus and vaccination, rather than on strategies for addressing callers’ existential, emotional, and mental difficulties. As a result, the HCs were largely on their own when handling emotional charged calls, which they sometimes found challenging and emotionally draining.

Theme 7: Lessons for future health crisis response

Building upon all previous themes, this final theme focuses on the key, hands-on lessons the HCs identified for operating a telephone service during a future pandemic. It also highlights broader lessons regarding the communication of health-related information more generally. First, the need for a multilingual telephone service became urgently apparent at the outset of the pandemic. Therefore, a recommendation for the future was to prioritize rapid establishment of a similar service in the event of a new pandemic. As demonstrated by the data presented above, the HCs stressed the importance of this service also being culturally informed and attuned to the structural challenges faced by migrant communities. Importantly, the HCs stressed that in a future pandemic, the telephone service would be especially valuable for newly arrived migrants, who often encounter particular challenges related to language, navigating the healthcare system, and interpreting information from government authorities.

A key limitation encountered by the HCs was the restriction against providing medical advice. For a future telephone service operating during a pandemic or a similar health crisis, they stressed the importance of including staff who are mandated to provide case-appropriate medical advice. Another significant limitation was the HCs need for better support in addressing the emotional problems and mental distress encountered among many callers. The HCs also wished they had been able to provide more effective psychological support themselves.

In Sweden, healthcare is managed by the regional governments. Another challenge was therefore operating a nationwide telephone service within a system where different Swedish regions applied varying regulations and procedures. For example, the specific routes to testing and vaccination would often differ between regions. Callers often needed practical guidance on what to do and where to turn, and the HCs found it difficult staying up-to-date with the various procedures in different parts of the country.

In sum, multilingual telephone services like the one evaluated could play an important role in future health crises. To maximize their effectiveness, future interventions should address a wider spectrum of caller needs, including psychological distress and the need for medical referrals.

**DISCUSSION**

**Structural and systemic barriers in access to care**

Our quantitative and qualitative findings highlight how structural and systemic conditions shaped patterns of access to the multilingual COVID-19 telephone service. The quantitative data showed that relatively few callers sought basic information about the virus or where to turn in case of illness, likely because the service was less well-known in the earliest phase of the pandemic when these issues were most urgent. In contrast, vaccination-related questions were more common later, once the service was more established. Notably, Somali speakers made up only 2.5% of callers, but when they did call, they more often requested information on where to seek medical care, and 87.5% of Somali-language calls were referred to the national healthcare telephone service “1177”. This suggests that this group may have needed personal medical advice more urgently, and often at later stages of illness. These patterns mirror mortality data showing that individuals born in Somalia (together with those born in Iraq and Syria) were disproportionately affected in terms of excess deaths in spring 2020 compared to Swedish-born individuals [2].

Such disparities must be seen against a broader backdrop of social determinants of health, including overcrowded housing, precarious employment, and experiences of stigma. The findings reported here underscores that crisis communication cannot be reduced to literal translations of majority-language information. Rather, public health messages must reflect and address relevant social, cultural, and structural realities of minoritized groups [20] and signal their inclusion in society during a health emergency. Alarmist media portrayals of affected migrant communities as “unsanitary others” [38] compounded these challenges. In Sweden, the Somali-speaking group in particular were affected by these negative media representations. Interviews with Swedish-Somali youth in a socioeconomically underprivileged neighborhood in Stockholm highlight feelings of enormous injustice associated with “being blamed for your own death” while struggling to uphold physical distancing in a context of overcrowded housing and precarious jobs [39].

**Trust, dialogue, and cultural brokerage**

Beyond the presence of structural barriers, our study shows that effective communication was rooted in relationships of trust. HCs underscored the importance of dialogue, consistent with the emphasis on a dialogue-based communication approach to successfully address vaccine hesitancy and resistance in other settings [40]. This echoes a broader shift toward ‘communicating with’ rather than ‘communicating to’ communities, so that populations targeted in public health outreach are also allowed to become engaged as stakeholders in designing and optimizing communication strategies. In that sense, dialogue-based approaches not only facilitated vaccine discussions but also affirmed the agency of vulnerable groups. In recent years, the importance of identifying ‘blind spots’ in mainstream public health campaigns has increasingly been stressed, in order to better tailor health communication with vulnerable groups that may not share underlying assumptions or find conventional health messages relevant to their contexts [41]. This paradigm shift has occurred against a backdrop of conventional public health communication in which health risk behaviors have typically been framed as individual and moral choices, often resulting in victim blaming and stigmatization [42,43]. Unfortunately, while public health advocates often point to the need to address sociocultural determinants of health, governments still tend to favor policy actions that aim to change individual-level behaviors through social marketing [44]. This is in spite of the known fact that people generally do not engage in health risk behaviors due to a lack of knowledge about risk, but because of life constraints that make them unable or unwilling to act differently [45]. More recently, previously accepted one-way models of health communication have been challenged, in favor of strategies that aim to uncover everyday contextualized experiences of health and illness and engage communities in a cooperative manner [46].

In the context of the multilingual telephone service, trust was actively co-produced through a combination of shared language, emic cultural, and professional affiliation with the Swedish healthcare system. This, for the HCs, seems to offer triangulation of the three key elements of language, culture, and professional competence in building trust with the callers. What emerges as particularly noteworthy, and perhaps counterintuitive given the widespread mistrust of authorities among many callers, is how professional healthcare expertise can actually enhance rather than undermine trust-building when it is delivered through culturally informed and linguistically accessible channels. Rather than professional authority creating distance, the HCs' ability to offer public health expertise through culturally resonant examples suggests that professional credibility and cultural sensitivity can work synergistically rather than in opposition to overcome institutional mistrust. In this role, HCs resembled ‘cultural brokers’, i.e., go-betweens mediating and translating between culturally distinct spheres [47]—in this case, this involved linguistic aspects as well as the different arenas inhabited by healthcare experts and lay people. Allowing callers to discuss their concerns with someone who shares lived experiences of migration contributes to a reciprocal communication process. Taken together, these findings suggest that trust was not simply a byproduct of providing accurate information, but the outcome of sustained relational and dialogical engagement.

**Information, misinformation, and credibility**

The COVID-19 pandemic also generated a massive parallel ‘infodemic’ of myths and conspiracy theories [48,49]. This includes poorly substantiated theories about the origin of COVID-19 and the peddling of fake cures. In this context, HCs played a key role in responding to misinformation. Their accounts indicate that anchoring conversations in available scientific knowledge, delivered in a culturally safe environment and native language, was particularly effective. For some callers, the opportunity to return and discuss new doubts further underscores the importance of continuity and credibility. These experiences align with so-called ‘inoculation models’ of health communication, which emphasize preemptive dissemination of accurate information and the cultivation of critical thinking [50]. More broadly, our findings point to the significance of credible messengers: scientific accuracy alone was insufficient unless paired with the relational trust that HCs had established.

**Psychosocial dimensions of crisis communication**

Not all calls were about factual information. Many calls were made in an obvious state of fear about the virus and its medical, social, and economic consequences, leaving individuals grappling with existential questions of life and death. Some callers phoned simply to talk, reflecting the pandemic’s profound psychosocial impact. The immediate impact of the COVID-19 pandemic on population-level mental health may have been slightly exaggerated [51]; even so, a meta-analysis suggests that there might have been more than a threefold increase in the rates of anxiety in the general population during the pandemic [52]. For survivors of COVID-19, persistent psychological problems including depression and anxiety have frequently been reported [53]. Among the reported risk factors for the development of anxiety were social isolation, unemployment, financial hardship, low education level, and insufficient knowledge of COVID-19. Furthermore, a low level of satisfaction with or trust in the measures taken by the government correlated with high anxiety scores.

This had implications not only for callers but also for staff. Telephone crisis support workers can experience vicarious traumatization and burnout, and they may not respond optimally to callers when experiencing elevated levels of distress [54]. Similarly, it can be challenging to address existential concerns in a highly secular society, such as Sweden [55,56]. In order to safeguard their well-being, the staff needs to be equipped with basic skills in crisis management as well as the opportunity to access support and guidance of their own. These findings echo those of other studies pointing to the importance of support for mitigating stress reactions among healthcare staff during future pandemics [57]. Overall, our study underscores that crisis communication should be understood as both an informational and a psychosocial intervention.

**Lessons for future health crisis response**

Several lessons emerge for future crisis preparedness. First, cooperation with local actors and organizations proved crucial. Even though the telephone service was national, the HCs worked to reach and engage with local civil society organizations. Religious congregations were considered particularly important. Again, such collaboration should take the form of a two-way exchange, including the sharing of resources and facilities and the joint tailoring of culturally and structurally relevant information to local communities. Religious leaders have proven to be valuable partners in disseminating information on COVID-19 restrictions and vaccination in other settings during the pandemic [58–60]. Moreover, they are uniquely equipped to address existential concerns, perhaps more so than any other profession. Faith communities within ethnic minority groups often have a longstanding history of mobilizing local resources and acting like safe havens in times of crisis [61]. Of course, there are also examples of religious leaders contributing in spreading misinformation and myths during the COVID-19 pandemic. If anything, this underscores the importance of engaging in dialogue with local religious institutions [62].

Second, access to continuous and up-to-date scientific support by relevant government agencies was vital. Managing a ‘mega-crisis’ such as the COVID-19 pandemic inevitably involves navigating unknown territories and adapting to new and rapidly changing circumstances [63]. The COVID-19 pandemic saw a fast-paced development of scientific knowledge and frequent modifications of guidelines and recommendations provided by authorities. Access to the best available scientific support, in the form of regular meetings with relevant government agencies, was crucial in keeping pace. Even so, an undue emphasis on the COVID-19 pandemic as uncharted waters may ignore the fact that several tried-and-tested public health interventions simply were not in place at the beginning of the pandemic. This includes, for example, a lack of pre-established collaborations with local civil society representatives to optimize efforts for reaching and disseminating information to migrant groups. The HCs staffing the multilingual COVID-19 telephone service thus had to combine the known-but-not-implemented and the new and unknown in their work.

Third, our findings caution against overreliance on digital communication. The strong current tendency in Swedish healthcare to rely on the internet and social media for the dissemination of health-related information risks becoming somewhat of a double-edged sword. In an evaluation of the handling of the COVID-19 pandemic, the Swedish Association of Local Authorities and Regions points to recently arrived immigrants as a group that has been exposed to digital exclusion, due to factors such as a lack of knowledge of the Swedish language and limited digital ability [64]. Similarly, our quantitative and qualitative data both show that a substantial number of calls concerned practical issues related to online healthcare solutions, such as how to book a vaccination appointment without electronic identification. Although a telephone service may seem like an old-fashioned option, many vulnerable groups do not have full access to newer online communication channels. The existence of individuals who rely on verbal information due to limited literacy is also often overlooked.

Integrating quantitative and qualitative results, we found that despite differences in caller languages—a majority of calls were conducted in Arabic and English with fewer calls in in Persian/Dari, Tigrinya, Russian, Somali, Amharic, Serbo-Croatian, and Spanish—the HCs described similar challenges and concerns across groups. This points to shared structural barriers rather than language- and culture-specific issues among callers as the primary drivers of calls. One interpretation of these findings is that while language and cultural competency were instrumental in building trust in the telephone service, the actual questions posed by the callers were mostly related to structural barriers. This is also reflected in the results from a study looking at direct and indirect effects of the COVID-19 pandemic on multiple social groups in Sweden, showing that the relative risk of being affected by negative events were strikingly similar across groups during the pandemic compared to the four years preceding it [65]. In effect, the socials determinants of health that disproportionally affected vulnerable groups in society before the pandemic were also the most important factors at play during the COVID-19 pandemic. This also means that there is not necessarily a need to reinvent the wheel. Culturally and structurally relevant health communication efforts that actively involve marginalized populations can clearly contribute to reducing the impact of health disparities, during a health crisis as well as in non-crisis times.

Taken together, our findings highlight the interdependence of structural conditions, dialogical trust-building, credible information delivery, and psychosocial support in effective health crisis communication. These results align with the theoretical framework for health crisis communication outlined by the CDC and others, emphasizing the phased, situation-specific, and culturally sensitive nature of effective outreach and community engagement with vulnerable populations [34,35]. More specifically, the HC experiences point to a lack of trust early on in the COVID-19 pandemic—in part related to common crisis psychology, but also aggravated by a lack of readily available culturally adapted information—that they were subsequently able to mitigate through their work with the multilingual telephone service. Importantly, while language and cultural competence were central to building trust, the substance of callers’ concerns often reflected persistent structural inequities. The implication is that inclusive, culturally and structurally attuned infrastructures for public health outreach should not be developed only in times of crisis but embedded in routine practice.

**Strengths and limitations**

The HCs participating in this study all had almost two years of experience in staffing the multilingual COVID-19 telephone service, enabling a unique insight into the possibilities and challenges associated with operating a crisis communication intervention for migrant groups. The aim of this study was to gain an unbiased insight into HC experiences of operating the telephone service and there was a great openness among the informants to share their experiences.

The findings presented here must also be viewed in light of a number of limitations. The study is based on the experiences of 12 HCs only. However, these 12 HCs had answered most of the 9 414 calls to the telephone line. It should also be noted that a qualitative study does not require a specific sample size in order to produce meaningful results [66]. In terms of the trustworthiness and rigor of the qualitative analysis, the fact that the HC experiences were based on a large number of telephone calls from all over Sweden covering almost the entire period of the pandemic supports the credibility of the findings. The thorough analysis process involving five researchers who reviewed the meaning units, themes, and subthemes several times until consensus was reached, as well as the use of the NVivo software, were ways to address rigor and reliability [67]. A weakness is that quantitative data on calls that concerned questions of a personal nature were removed by the PHAS for reasons of confidentiality, limiting our possibilities to draw more detailed conclusions regarding worries related to personal medical issues and referrals to the national healthcare telephone service “1177”. An overall limitation is that the nature of the available data does not allow us to analyze precisely which groups were actually reached by the telephone service and which were not, beyond the statistics on the preferred language of the callers. Yet another limitation is that it was not possible to use a prospective study design, since the multilingual telephone line was set up in an emergency pandemic situation. Finally, although the study was not designed to evaluate the performance of the HCs, there is a possibility that some staff experiences might not have been shared for fear of reflecting poorly on the telephone service.

**Conclusion and implications**

Experiences from a multilingual telephone service in Sweden point to the value of health crisis communication that offers the possibility of dialogue with public health professionals in a culturally safe mode using one's native language. The results highlight the importance of linguistic, cultural, and structural competence for communicating and disseminating relevant information and building trust in times of a health crisis. Moreover, for future research, the experiences from this study point to the necessity of making use of the (often retrospective) data that are possible to collect in a naturalistic pandemic emergency setting. Our findings emphasize the importance of establishing communication strategies that target vulnerable groups *before* the need arises, as part of a comprehensive pandemic plan. This is particularly important for linguistically and socioculturally diverse communities in which the uptake of public health information created with the majority population in mind cannot be taken for granted. Moreover, the ability of marginalized groups to navigate the healthcare system can be strengthened by establishing dialogue and cooperation with civil society organizations and other local representatives of migrant communities. Sufficient support structures providing up-to-date scientific information must be available. However, health crisis communication interventions such as a telephone service should also be able to accommodate emotional reactions, existential concerns, and mental distress among callers. The HC experiences have the potential to contribute important knowledge about vital aspects of multilingual crisis communication relevant to settings wider than a pandemic.

**List of abbreviations**

CDC: United States Centers for Disease Control and Prevention

COVID-19: Coronavirus disease 2019

PCR: Polymerase chain reaction

PHAS: Public Health Agency of Sweden

RR: Relative risk

WHO: World Health Organization

**Ethics approval and consent to participate**

The study was approved by the Swedish Ethical Review Authority (No. 2022-01637-01). Written consent was obtained from all participants.

**Consent for publication**

Not applicable.

**Availability of data and materials**

The datasets used during the current study are available from the corresponding author on reasonable request.

**Competing interests**

The authors declare that they have no competing interests.

**Funding**

The current study is funded by the Swedish Research Council (reference number 2021-06276).

**Authors’ contribution**

Sofie Bäärnhielm: Conceptualization; Data curation; Formal analysis; Funding acquisition; Investigation; Methodology; Project administration; Resources; Software; Supervision; Validation; Writing - original draft; Writing - review & editing.

Baidar Al-Ammari: Data curation; Formal analysis; Investigation; Writing - review & editing.

Önver Cetrez: Conceptualization; Investigation; Methodology; Writing – review & editing.

Soorej Jose Puthoopparambil: Conceptualization; Investigation; Methodology; Visualization; Writing - review & editing

Mattias Strand: Conceptualization; Data curation; Formal analysis; Investigation; Methodology; Writing - original draft; Writing - review & editing

**Acknowledgments**

We want to thank the participating health communicators for sharing their experiences and the Public Health Agency of Sweden for sharing quantitative data.

**REFERENCES**

1. World Health Organization. COVID-19 Response in the World Health Organization African Region, February to December 2021. Brazzaville, CG: WHO Regional Office for Africa; 2021.

2. Hansson E, Albin M, Rasmussen M, Jakobsson K. Stora skillnader i överdödlighet våren 2020 utifrån födelseland. Lakartidningen. 2020;117:28–32.

3. Yaya S, Yeboah H, Charles CH, Otu A, Labonte R. Ethnic and racial disparities in COVID-19-related deaths: counting the trees, hiding the forest. BMJ Glob Heal. 2020;5:e002913.

4. Irizar P, Pan D, Kapadia D, Bécares L, Sze S, Taylor H, et al. Ethnic inequalities in COVID-19 infection, hospitalisation, intensive care admission, and death: a global systematic review and meta-analysis of over 200 million study participants. eClinicalMedicine. 2023;57. doi.org/10.1016/j.eclinm.2023.101877

5. Marmot M, Allen J, Goldblatt P, Herd E, Morrison J. Build Back Fairer: The COVID-19 Marmot Review. The Pandemic, Socioeconomic and Health Inequalities in England. London, UK: UCL Institute of Health Equity; 2020.

6. Rostila M, Cederström A, Wallace M, Aradhya S, Ahrne M, Juárez SP. Inequalities in COVID-19 severe morbidity and mortality by country of birth in Sweden. Nat Commun. 2023;14:4919. doi.org/10.1038/s41467-023-40568-4

7. Myndigheten för vård- och omsorgsanalys. Riktade vaccinationsinsatser: Lärdomar från regionernas arbete för en hög och jämlik vaccinationstäckning mot covid-19. Stockholm, SE: Myndigheten för vård- och omsorgsanalys; 2022.

8. Khunti K, Singh AK, Pareek M, Hanif W. Is ethnicity linked to incidence or outcomes of covid-19? BMJ. 2020;369:m1548

9. Kluge HHP, Jakab Z, Bartovic J, D’Anna V, Severoni S. Refugee and migrant health in the COVID-19 response. Lancet. 2020;395:1237–9. doi.org/10.1016/S0140-6736(20)30791-1

10. Page KR, Venkataramani M, Beyrer C, Polk S. Undocumented U.S. Immigrants and Covid-19. N Engl J Med. 2020;382:e62. doi.org/10.1056/NEJMp2005953

11. Hitch L, Masoud D, Hobbs LA, Moujabber M, Cravero K. The vulnerability to COVID-19 of migrants in large urban areas: structural exacerbators and community-level mitigators. Eur J Public Health. 2023;33:704–16.

12. Martínez-Donate AP, Correa-Salazar C, Bakely L, González-Fagoaga JE, Asadi-Gonzalez A, Lazo M, et al. COVID-19 testing, infection, and vaccination among deported Mexican migrants: Results from a survey on the Mexico-U.S. border. Front Public Heal. 2022;10:928385.

13. Söderberg M, Cronie O, Adiels M, Rosengren A. The influence of overcrowding and socioeconomy on the spatio-temporal spread of COVID-19 - a Swedish register study. Göteborg, SE: Göteborg University; 2022.

14. Nutbeam D, Lloyd JE. Understanding and Responding to Health Literacy as a Social Determinant of Health. Annu Rev Public Health. 2021;42:159–73. doi.org/10.1146/annurev-publhealth-090419-102529

15. Wångdahl J, Lytsy P, Mårtensson L, Westerling R. Health literacy and refugees’ experiences of the health examination for asylum seekers – a Swedish cross-sectional study. BMC Public Health. 2015;15:1162. doi.org/10.1186/s12889-015-2513-8

16. Burns R, Campos-Matos I, Harron K, Aldridge RW. COVID-19 vaccination uptake for half a million non-EU migrants and refugees in England: a linked retrospective population-based cohort study. Lancet. 2022;400:S5.

17. Fernández-Sánchez H, Zahoui Z, Jones J, Marfo EA. Access, acceptability, and uptake of the COVID-19 vaccine among global migrants: A rapid review. PLoS One. 2023;18:e0287884.

18. Page KR, Genovese E, Franchi M, Cella S, Fiorini G, Tlili R, et al. COVID-19 vaccine hesitancy among undocumented migrants during the early phase of the vaccination campaign: a multicentric cross-sectional study. BMJ Open. 2022;12:e056591.

19. Lin S. COVID-19 Pandemic and Im/migrants’ Elevated Health Concerns in Canada: Vaccine Hesitancy, Anticipated Stigma, and Risk Perception of Accessing Care. J Immigr Minor Heal. 2022;24:896–908.

20. Ortega P, Martínez G, Diamond L. Language and Health Equity during COVID-19: Lessons and Opportunities. J Health Care Poor Underserved. 2020;31:1530–5.

21. Nordic Council of Ministers. Outreach and dissemination of public information to immigrants during the COVID-19 pandemic. Copenhagen, DK: Nordic Council of Ministers; 2022.

22. Hansson E, Jakobsson K. Covid-19 i trångbodda förorter och på äldreboende - samverkande strukturella faktorer? En geografisk analys av samband mellan förutsättningar för social distans och kontakter med äldre i Stockholm, Göteborg och Malmö. Göteborg, SE: Göteborg University; 2020.

23. World Health Organization. ApartTogether survey: Preliminary overview of refugees and migrants self-reported impact of COVID-19. Geneva, CH: WHO; 2020.

24. Folkhälsomyndigheten. Hur har folkhälsan påverkats av covid-19-pandemin? Samlad bedömning utifrån svensk empiri och internationell forskning under 2020. Stockholm, SE: Folkhälsomyndigheten; 2021.

25. Esaiasson P, Johansson B, Ghersetti M, Sohlberg J. Kriskommunikation och segregation i en pandemi: Hur boende i utsatta områden informerade sig om coronaviruset våren 2020. Göteborg, SE: Göteborg University; 2020.

26. Cai D-Y. A concept analysis of cultural competence. Int J Nurs Sci. 2016;3:268–73.

27. Metzl JM, Hansen H. Structural competency: Theorizing a new medical engagement with stigma and inequality. Soc Sci Med. 2014;103:126–33.

28. Ahearn LM. Language and Agency. Annu Rev Anthropol. 2001;30:109–37. doi.org//10.1146/annurev.anthro.30.1.109

29. Bresnahan M, Zhuang J. Culturally safe healthcare: changing the lens from provider control to patient agency. J Commun Healthc. 2024;17:244–53.

30. Statistiska centralbyrån. Statistikdatabasen. Örebro, SE: SCB; 2022.

31. Björkman A, Gisslén M, Gullberg M, Ludvigsson J. The Swedish COVID-19 approach: a scientific dialogue on mitigation policies. Front Public Heal. 2023;11. doi.org/10.3389/fpubh.2023.1206732

32. Smittskydd Stockholm. Epidemiberedskapsplan, Region Stockholm. Stockholm, SE: Smittskydd Stockholm; 2019.

33. Bäärnhielm S, Al-Ammari B, Hussein H. Erfarenheter från Region Stockholms telefonlinje om covid-19 på olika språk: Interkulturell kommunikation i samarbete mellan vård och civilsamhälle. Soc Tidskr. 2021;124–30.

34. United States Centers for Disease Control and Prevention. Crisis & Emergency Risk Communication (CERC) Manual. Atlanta, GA: CDC; 2018.

35. Vaughan E, Tinker T. Effective Health Risk Communication About Pandemic Influenza for Vulnerable Populations. Am J Public Health. 2009;99:S324–32. doi.org/10.2105/AJPH.2009.162537

36. Braun V, Clarke V. Thematic Analaysis: A Practical Guide. Thousand Oaks, CA: SAGE Publications; 2021.

37. Kvale S. InterViews: An introduction to qualitative research interviewing. InterViews: An Introduction to Qualitative Researh Interviewing. Thousand Oaks, CA: Sage Publications; 1994.

38. Desmarais C, Roy M, Nguyen MT, Venkatesh V, Rousseau C. The unsanitary other and racism during the pandemic: analysis of purity discourses on social media in India, France and United States of America during the COVID-19 pandemic. Anthropol Med. 2023;30:31–47. doi.org/10.1080/13648470.2023.2180259

39. Abdi N. “Somalierna fick skulden för sin egen död”: En kvalitativ studie av hur somalier i Järvområdets upplevt medierapporteringen under Coronapandemin. Uppsala, SE: Uppsala University; 2021.

40. Peters MDJ. Addressing vaccine hesitancy and resistance for COVID-19 vaccines. Int J Nurs Stud. 2022;131:104241.

41. Dutta-Bergman MJ. Theory and Practice in Health Communication Campaigns: A Critical Interrogation. Health Commun. 2005;18:103–22. doi.org/10.1207/s15327027hc1802_1

42. Hanlon P, Carlisle S, Hannah M, Reilly D, Lyon A. Making the case for a “fifth wave” in public health. Public Health. 2011;125:30–6.

43. Brewis A, Wutich A. Lazy, Crazy, and Disgusting: Stigma and the Undoing of Global Health. Baltimore, MD: Johns Hopkins University Press; 2019.

44. Short SE, Mollborn S. Social Determinants and Health Behaviors: Conceptual Frames and Empirical Advances. Curr Opin Psychol. 2015;5:78–84.

45. Baum F, Fisher M. Why behavioural health promotion endures despite its failure to reduce health inequities. Sociol Health Illn. 2014;36:213–25. doi.org/10.1111/1467-9566.12112

46. Zoller HM, Kline KN. Theoretical Contributions of Interpretive and Critical Research in Health Communication. Ann Int Commun Assoc. 2008;32:89–135. doi.org/10.1080/23808985.2008.11679076

47. Miklavcic A, LeBlanc MN. Cultural Brokers, Clinical Applied Ethnography, and Cultural Mediation. In: Kirmayer LJ, Guzder J, Rousseau C, editors. Cultural Consultation: Encountering the Other in Mental Health Care. New York, NY: Springer Nature; 2014. p. 115–37.

48. Mheidly N, Fares J. Leveraging media and health communication strategies to overcome the COVID-19 infodemic. J Public Health Policy. 2020;41:410–20. doi.org/10.1057/s41271-020-00247-w

49. Chong YY, Cheng HY, Chan HYL, Chien WT, Wong SYS. COVID-19 pandemic, infodemic and the role of eHealth literacy. Int J Nurs Stud. 2020;108:103644.

50. van der Linden S, Roozenbeek J, Compton J. Inoculating Against Fake News About COVID-19. Front Psychol. 2020;11:566790.

51. Ahmed N, Barnett P, Greenburgh A, Pemovska T, Stefanidou T, Lyons N, et al. Mental health in Europe during the COVID-19 pandemic: a systematic review. Lancet Psychiatry. 2023;10:537–56. doi.org/10.1016/S2215-0366(23)00113-X

52. Santabárbara J, Lasheras I, Lipnicki DM, Bueno-Notivol J, Pérez-Moreno M, López-Antón R, et al. Prevalence of anxiety in the COVID-19 pandemic: An updated meta-analysis of community-based studies. Prog Neuro-Psychopharmacology Biol Psychiatry . 2021;109:110207.

53. Fahriani M, Ilmawan M, Fajar JK, Maliga HA, Frediansyah A, Masyeni S, et al. Persistence of long COVID symptoms in COVID-19 survivors worldwide and its potential pathogenesis - a systematic review and meta-analysis. Narra J. 2021;1:e36.

54. Kitchingman TA, Wilson CJ, Caputi P, Wilson I, Woodward A. Telephone Crisis Support Workers’ Psychological Distress and Impairment. Crisis. 017;39:13–26. doi.org/10.1027/0227-5910/a000454

55. Nissen RD, Andersen AH. Addressing Religion in Secular Healthcare: Existential Communication and the Post-Secular Negotiation. Religions. 2022;13:34.

56. Nygaard MR, Austad A, Sørensen T, Synnes O, McSherry W. ‘Existential’ in Scandinavian Healthcare Journals: An Analysis of the Concept and Implications for Future Research. Religions. 2022;13:979.

57. Hamdan A, Eastaugh J, Snygg J, Naidu J, Alhaj I. Coping strategies used by healthcare professionals during COVID-19 pandemic in Dubai: A descriptive cross-sectional study. Narra X. Narra T; 2023;1.

58. Monson K, Oluyinka M, Negro D, Hughes N, Maydan D, Iqbal S, et al. Congregational COVID-19 Conversations: Utilization of Medical-Religious Partnerships During the SARS-CoV-2 Pandemic. J Relig Health. 2021;60:2353–61.

59. Wijesinghe MSD, Ariyaratne VS, Gunawardana BMI, Rajapaksha RMNU, Weerasinghe WMPC, Gomez P, et al. Role of religious leaders in covid-19 prevention: A community-level prevention model in Sri Lanka. J Relig Health. 2022;61:687–702.

60. Kasstan B, Mounier-Jack S, Gaskell KM, Eggo RM, Marks M, Chantler T. “We’ve all got the virus inside us now”: Disaggregating public health relations and responsibilities for health protection in pandemic London. Soc Sci Med. 2022;309:115237.

61. Bruce MA. COVID-19 and African American Religious Institutions. Ethn Dis. 2020;30(3):425–8.

62. Kasstan B. Vaccines and vitriol: an anthropological commentary on vaccine hesitancy, decision-making and interventionism among religious minorities. Anthropol Med. 2021;28:411–9. doi.org/10.1080/13648470.2020.1825618

63. Lagadec P. The Unknown Territory of Mega-Crisis: In Search of Conceptual and Strategic Breakthroughs. In: Helsloot I, Boin A, Jacobs B, Comfort LK, editors. Mega-Crises: Understanding the Prospects, Nature, Characteristics and Effects of Cataclysmic Events. Springfield, IL: Charles C Thomas Publisher; 2012. p. 12–24.

64. Sveriges Kommuner och Regioner. Att lära av en kris: Kommuners och regioners lärdomar från covid-19-pandemin. Stockholm, SE: SKR; 2023.

65. Altmejd A, Östergren O, Björkegren E, Persson T. Inequality and COVID-19 in Sweden: Relative risks of nine bad life events, by four social gradients, in pandemic vs. prepandemic years. Proc Natl Acad Sci. 2023;120:e2303640120. doi.org/10.1073/pnas.2303640120

66. Busetto L, Wick W, Gumbinger C. How to use and assess qualitative research methods. Neurol Res Pract. 2020;2:14. doi.org/10.1186/s42466-020-00059-z

67. Graneheim UH, Lindgren B-M, Lundman B. Methodological challenges in qualitative content analysis: A discussion paper. Nurse Educ Today. 2017;56:29–34.
